# Intramolecular H−Migration Kinetics of •OOQOOH Radicals for KHP Formation During Low-Temperature Oxidation of Alkylcyclohexanes

**DOI:** 10.3390/molecules31183299

**Published:** 2026-09-17

**Authors:** Xiaoxia Yao, Yuheng Liu, Ying Xuan, Junjiang Guo, Mingxia Liu, Zerong Li, Wenjiong Hai

**Affiliations:** 1 Aviation Maintenance Industry College, Chengdu Aeronautic Polytechnic University, Chengdu 610100, China; 2 School of Chemical Engineering, Guizhou Institute of Technology, Guiyang 550003, China; 3 School of Physics and New Energy, Chongqing University of Technology, Chongqing 401135, China; 4 College of Chemistry, Sichuan University, Chengdu 610064, China

**Keywords:** alkylcyclohexane, •OOQOOH radical, intramolecular H−migration, quantum chemical calculation, low−temperature oxidation

## Abstract

Alkylcyclohexanes are vital components of aviation kerosene. Intramolecular H−migration of •OOQOOH radicals controls ketohydroperoxide (KHP) formation, the primary pathway responsible for low-temperature chain-branching during low−temperature oxidation. Available chemical kinetic models for alkylcyclohexanes generally lack directly computed kinetic data for H−migration reactions of •OOQOOH in cyclic fuels; relevant rate constants are commonly transferred from analogous alkane reactions, introducing systematic uncertainties in low−temperature ignition predictions. In this work, quantum chemical calculations are performed for 13 representative •OOQOOH intramolecular H−migration pathways originating from alkylcyclohexanes, covering six structural subclasses: 1,5−H−(s)(p), 1,5−H−(s)(s), 1,5−H−(t)(p), 1,5−H−(t)(s), 1,6−H−(t)(p), and 1,6−H−(t)(s). Modified Arrhenius parameters are fitted from high−pressure−limit rate constants over 500–1500 K. Further comparison between the present computed rate data and parameters adopted in existing mechanisms demonstrates that the literature values transferred from alkanes are systematically lower. Ring strain and distinct transition−state entropy originating from cyclic structures make the alkane kinetic parameters inappropriate for alkylcyclohexane systems. The kinetic parameters and lumped subclass rate rules obtained in this study provide fundamental data for improving low-temperature oxidation models of alkylcyclohexanes.

## 1. Introduction

Cycloalkanes [1,2] are essential components of fuels such as diesel and aviation kerosene. Their volume fraction can reach 20% in Jet-A fuel and even exceed 50% in certain shale oils and biofuels [3,4,5,6,7,8]. Furthermore, cycloalkanes are frequently selected as one of the constituent species in surrogate fuels for investigations into the combustion and oxidation characteristics of diesel and aviation kerosene [9,10,11]. Consequently, a thorough understanding of their combustion behavior and underlying kinetic mechanisms is essential for improving engine combustion efficiency and curbing pollutant emissions [12]. Despite growing advances in the combustion chemistry of cyclic hydrocarbons over recent years, high-precision kinetic data for long-chain linear alkylcyclohexanes, including methylcyclohexane, ethylcyclohexane, n−propylcyclohexane, and n−butylcyclohexane, remain far less comprehensive than those well-established for conventional acyclic alkanes. Distinct from straight−chain alkanes, the rigid cyclohexane skeleton enables unique reaction pathways such as ring-opening reactions, which substantially complicate the overall reaction network and yield more intricate kinetic schemes [13].

It is widely recognized that hydrocarbon fuels oxidize via fundamentally different pathways at low and high temperatures [1,14]. At high temperatures, alkyl radicals are primarily channeled into *β*−scission due to the high barriers involved. At low temperatures, combustion initiation involves H-abstraction from fuel molecules to generate carbon-centered radicals [15,16], which subsequently react with O_2_ to form peroxyalkyl radicals (ROO•). Intramolecular H migration within ROO• produces hydroperoxyalkyl radicals (•QOOH) [13,17,18,19,20,21,22], followed by a second O_2_ addition at the radical site of •QOOH to yield hydroperoxyalkylperoxy radicals (•OOQOOH). The ring size of the transition state (TS) for ROO• H migration dictates the spatial separation between −OO• and -OOH moieties in •OOQOOH [23]. •OOQOOH also undergoes intramolecular H migration, in which a hydrogen atom from a C-H bond within the molecule migrates to the terminal oxygen of the peroxy radical (−OO•), generating a new hydroperoxy group and a carbon-centered radical [17,18,23,24]. If the migrating H is located on the *α*−carbon relative to the -OO moieties, this process generates ketohydroperoxide (KHP) and an OH radical [24]. Cleavage of the O−OH bond in KHP releases another OH radical and an oxy radical with an sp^3^-hybridized carbon center.

Current kinetic models for alkylcyclohexanes often overlook or oversimplify •OOQOOH H-migration reactions due to the lack of accurate rate constants. Even in mechanisms that include these reactions, kinetic parameters are typically extrapolated from analogous reactions in acyclic alkanes or from ROO• H-migration data in cyclohexanes. For example, Liu et al. [9] constructed a detailed kinetic model for n−propylcyclohexane combustion based on AramcoMech 3.0, where rate constants for •OOQOOH H-migration (forming •P(OOH)_2_ or KHP + OH) were approximated using ROO• H-migration kinetics from prior n-propylcyclohexane studies [13]. Based on the collaborative model proposed by Natelson et al., Mao et al. [10] constructed a mechanism to model the ignition behavior of n−butylcyclohexane. In this mechanism, the high pressure−limit rate constants for the intramolecular H migration of •OOQOOH that yields KHP and an OH radical were estimated by analogy to analogous alkane reactions. Similarly, in their subsequent low-temperature combustion mechanism for n-butylcyclohexane [11], the same group adopted rate constants for the •OOQOOH → KHP + OH pathway directly from corresponding alkane reactions. Our previous work [13] demonstrated that analogous reactions in alkylcyclohexanes and acyclic alkanes exhibit significant kinetic differences, rendering alkane-derived parameters unsuitable for cyclohexane systems.

The present work aims to systematically explore the kinetics of •OOQOOH intramolecular H−migration pathways generating KHP and OH radicals over a wide temperature range. Quantum chemical calculations are carried out for a series of typical reaction channels in alkylcyclohexanes. The kinetic parameters obtained herein help reveal the cyclic structural effects on reaction barriers, and supply fundamental data to support the automated development of low-temperature oxidation mechanisms for alkylcyclohexanes.

## 2. Discussion

### 2.1. Potential Energy Surfaces

At temperatures below 800 K, approximately 95% of cyclohexane molecules exist as chair conformers. As reported in previous studies [1,25], the energy gap between the chair conformer and the twist-boat conformer (the second most stable structure) is 19.6–25.9 kJ mol^−1^, which confirms the superior thermodynamic stability of the chair geometry. Accordingly, the favorable chair conformers associated with primary H-migration reactions are adopted in this work to calculate the reaction pathways of the second oxygen addition step. Although both equatorial and axial hydrogens of chair cyclohexane are accessible for alkyl substitution, numerous previous studies [25,26] have demonstrated that alkyl side chains thermodynamically prefer equatorial orientations. To quantitatively characterize the energy differences in methyl and ethyl substituents at these two spatial sites, molecular systems with axial and equatorial methyl/ethyl groups are constructed for methylcyclohexane and ethylcyclohexane, and corresponding energy calculations are carried out in this work. The results are presented in Table 1.

As listed in Table 1, conformers with equatorial alkyl substituents exhibit more negative electronic energies and superior thermodynamic stability for both methylcyclohexane and ethylcyclohexane. The energy difference between axial and equatorial geometries reaches almost 1.9 kcal mol^−1^ for both molecules. Axial alkyl groups introduce severe 1,3−diaxial steric repulsion, while equatorial placement greatly alleviates intramolecular van der Waals strain, leading to the universal equatorial preference of alkyl side chains on cyclohexane rings. Accordingly, this work only investigates the chemical kinetics of intramolecular H migration after the second oxygen addition for chair cyclohexane molecules substituted with linear alkyl side chains (methyl, ethyl, n−propyl, n−butyl).

The •OOQOOH are generated by an O_2_ addition to the radical sites of •QOOH, which are produced by intramolecular H−migration of ROO•. Therefore, the configuration of the •OOQOOH is determined by the configuration of •QOOH. Theoretical and modeling studies on alkylcyclohexanes by Knepp et al. [27], Xing et al. [28,29], Serinyel et al. [30], Weber et al. [31], and Bissoonauth et al. [32] have shown that 1,5 H−migration dominates the intramolecular H−migration of ROO•. Hence, Zou et al. [33] considered the •QOOH generated through the 1,5 H−migration of ROO• as a primary intermediate for subsequent oxygenation in their development of a low-temperature combustion mechanism for ethylcyclohexane.

**Scheme 1:**
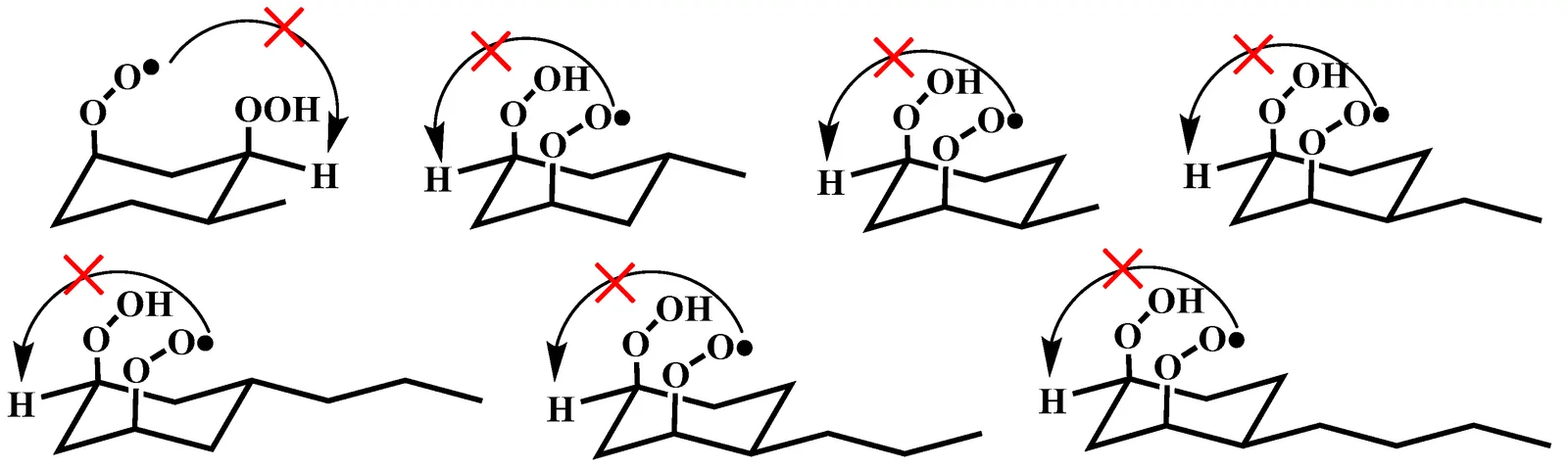
Cases of impossible H−migration for •OOQOOH with both −OO• and −OOH moieties on the ring arising from steric hindrance [10,33]. (The red cross denotes that this reaction pathway cannot occur).

In addition, the chair conformation of the six−membered cycle is selected as part of the research system in this study, and the positions of the -OO• and -OOH moieties in the reactant •OOQOOH tend to be perpendicular to the chair conformation, as shown in Scheme 1 [10,33]. From Scheme 1, it can be observed that when the carbon sites of the six−membered cycle have both −OO• and −OOH moieties, •OOQOOH will be affected by a steric hindrance effect if it wants to migrate a hydrogen atom from the carbon atom where the −OOH moiety is located to produce KHP + •OH products, so that the above reaction pathway cannot occur. Therefore, in the present work, the pathways that involve intramolecular H−migration of •OOQOOH with both OO• and OOH moieties on the cycle carbon sites are not considered, and this class of reactions can occur only if at least one of the OOH and OO• moieties is on the alkyl side chain.

**Scheme 2:**
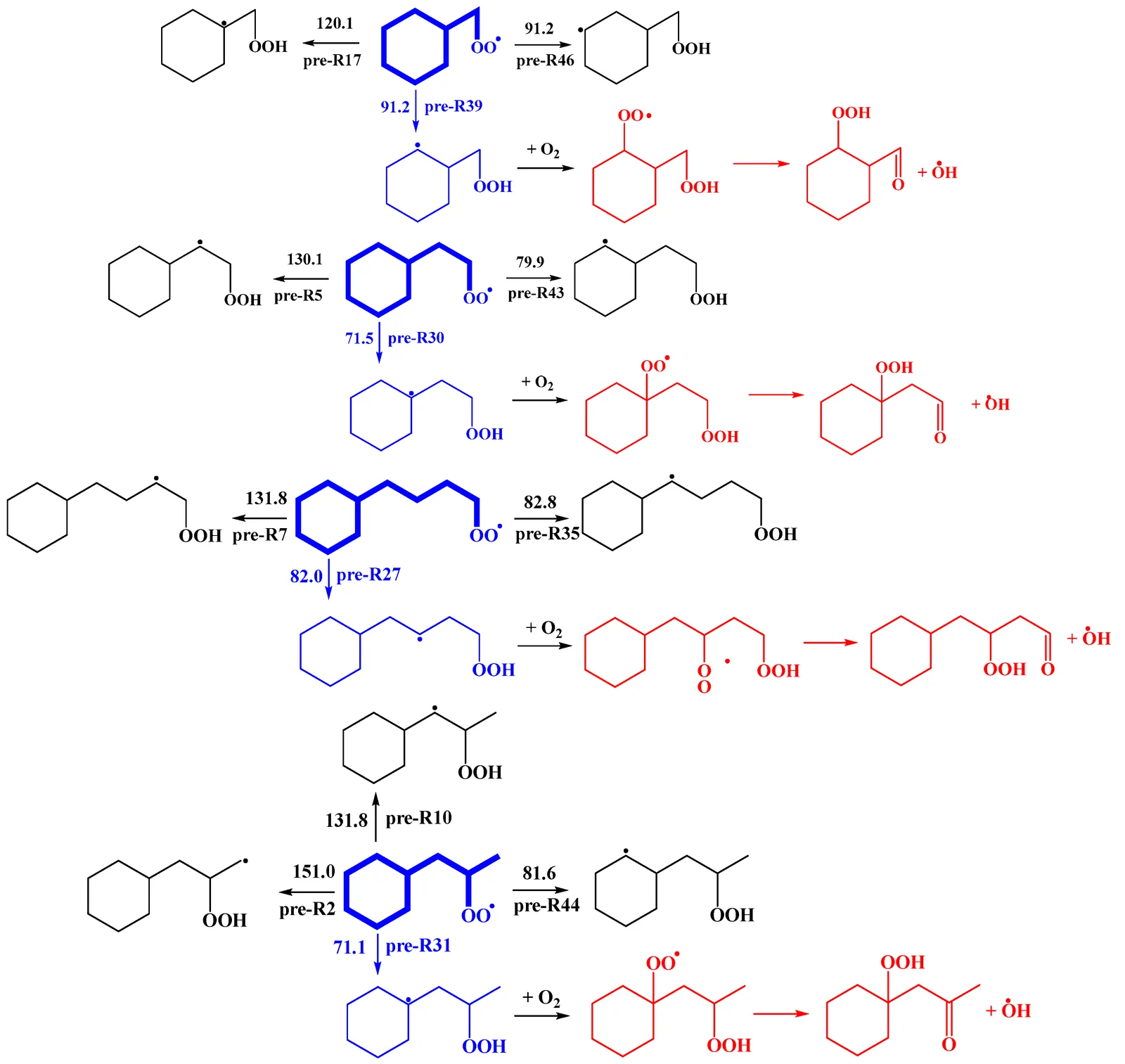

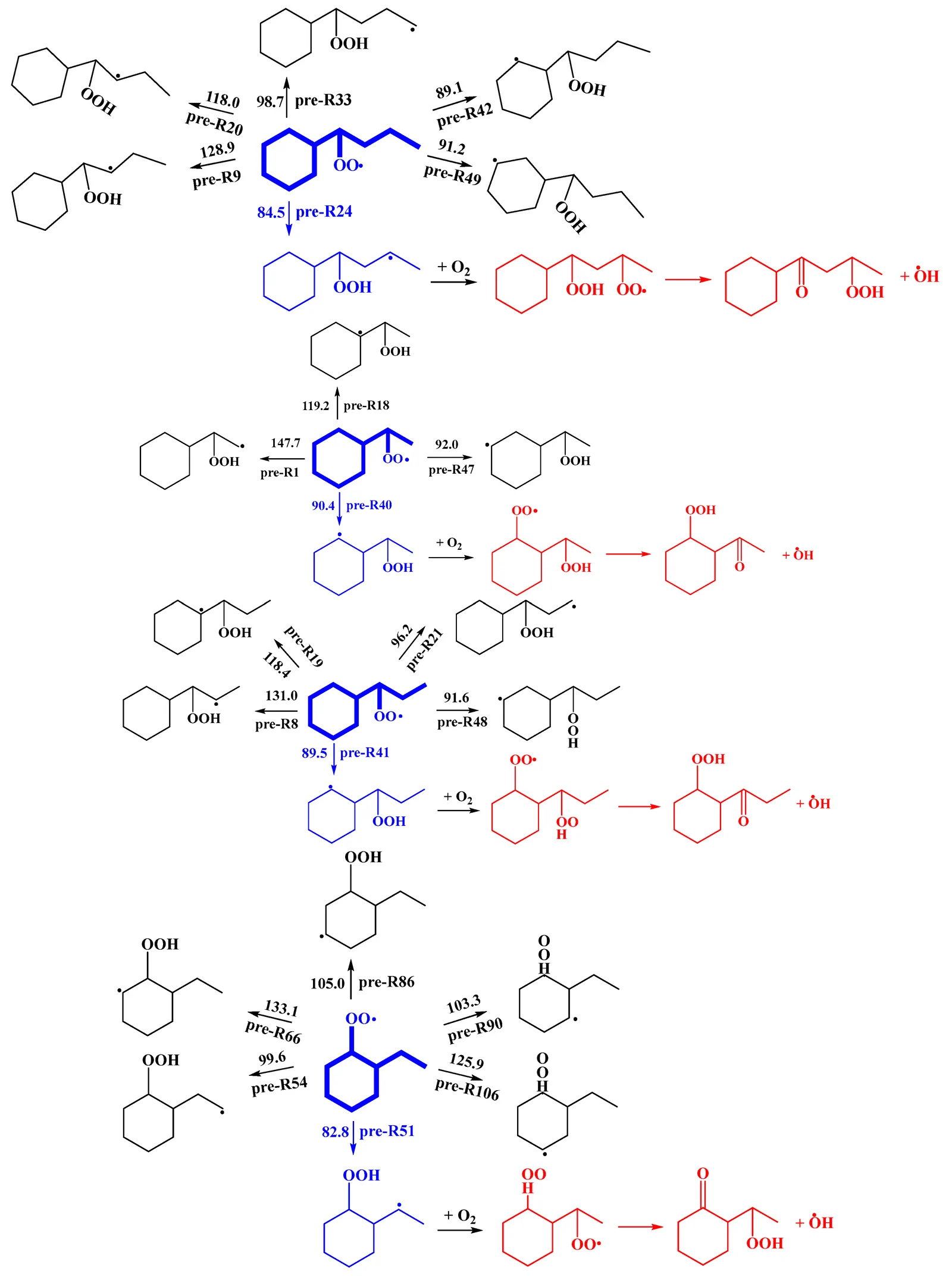

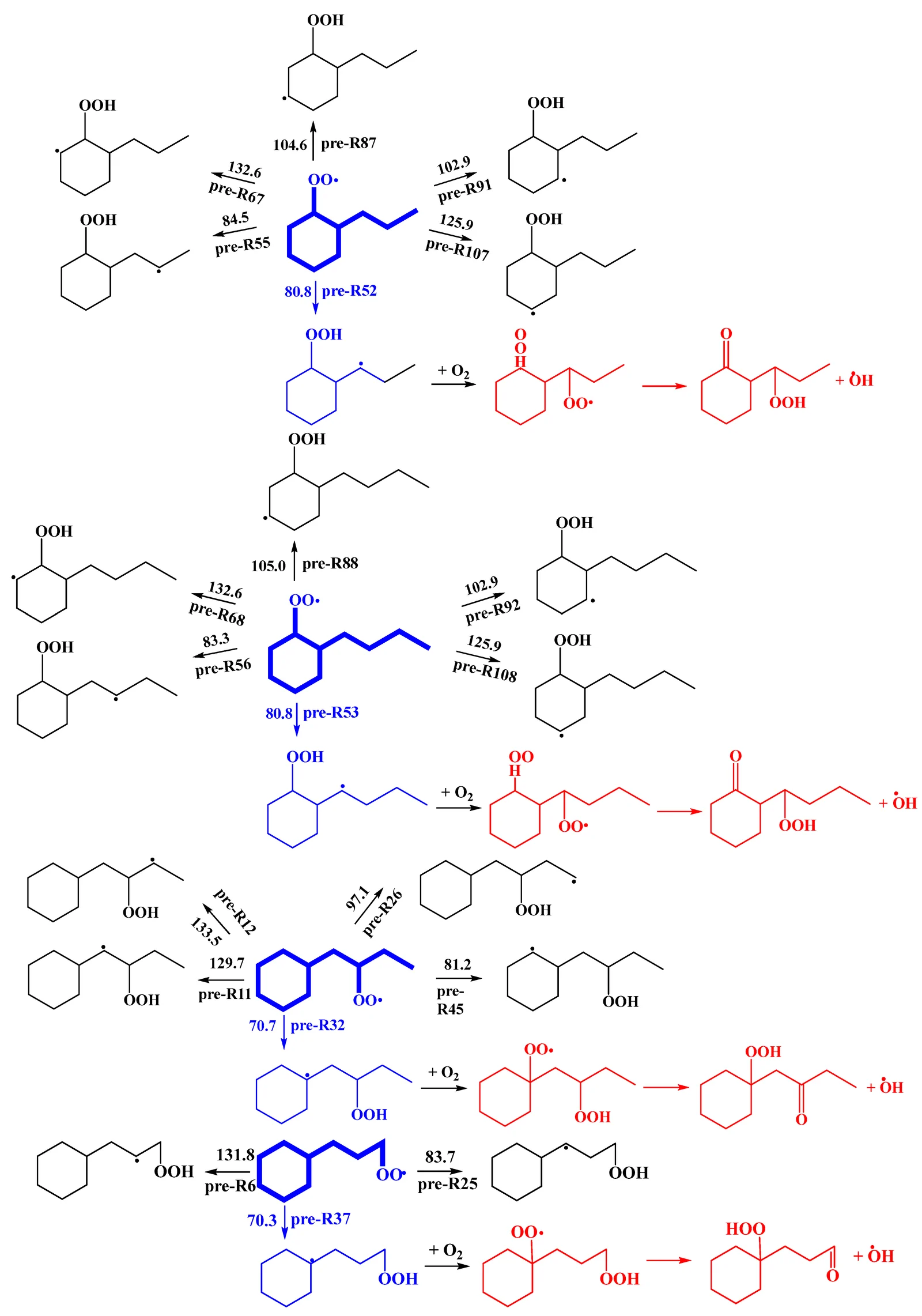

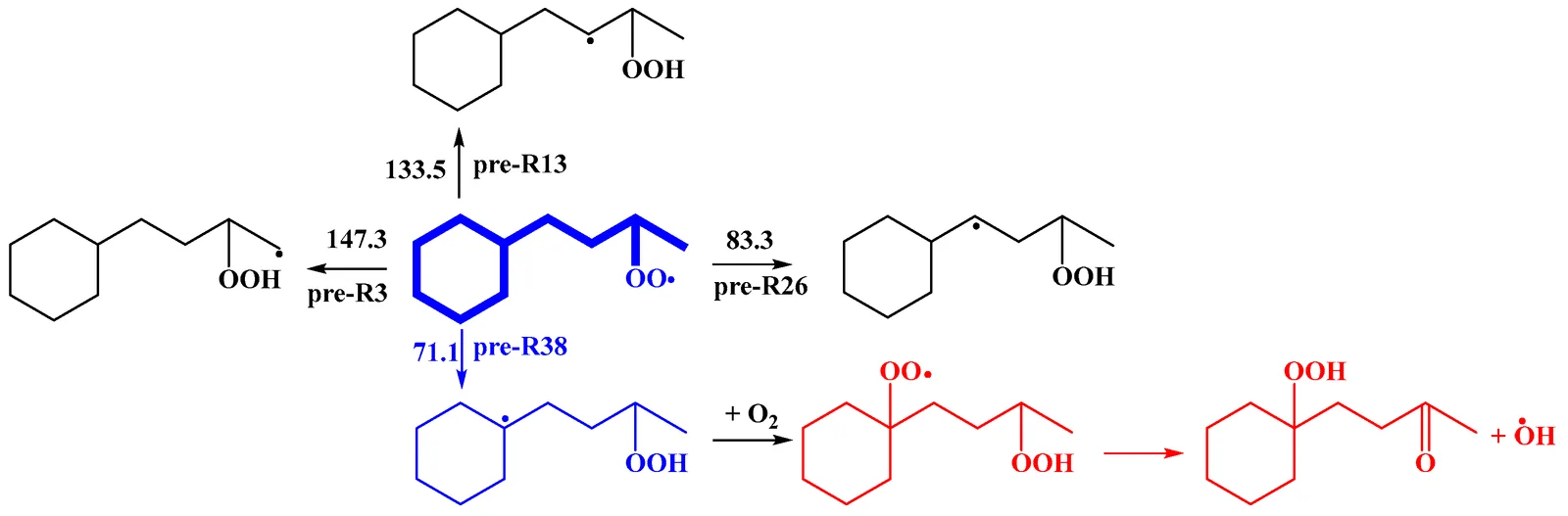
Reaction sequence: ROO• → •QOOH→ •OOQOOH → products. Reactions highlighted in blue were investigated in our previous work [13], whereas those highlighted in red represent the reactions investigated in the present work. Energy values are given in kJ mol^−1^.

The low-temperature oxidation pathways of alkylcyclohexanes proceed via successive O_2_ addition to carbon-centered radicals. As illustrated in Scheme 2, the reaction channels highlighted in blue correspond to the intramolecular H−migration reactions of cycloalkylalkyl radicals formed after the first O_2_ addition, which have been systematically investigated in our previous work [13]. After the initial O_2_ addition, the generated peroxyl radical undergoes intramolecular H−migration to produce a new carbon-centered radical bearing the −OOH moiety. This radical can capture a second O_2_ molecule to form •OOQOOH radicals (structures marked in red), which are central species controlling low-temperature chain-branching.

To provide a clear overview of the reaction pathways considered in this work, the low-temperature oxidation sequence leading to KHP formation is schematically illustrated in Scheme 2. As shown in the scheme, ROO• radicals undergo intramolecular H−migration to form •QOOH radicals, followed by a second O_2_ addition to generate •OOQOOH intermediates. Subsequent intramolecular H−migration and decomposition of •OOQOOH lead to the formation of KHP + •OH. In our previous work [13], several intramolecular H−migration pathways of ROO• radicals following the first O_2_ addition were investigated. The pathway highlighted in blue in Scheme 2 corresponds to the reaction with the lowest calculated barrier among those examined in that study. In the present work, the product formed through this lowest-barrier pathway was selected as the precursor for the subsequent second O_2_ addition, and the resulting •OOQOOH species were then used to investigate the second-stage intramolecular H−migration reactions. These target reactions are highlighted in red in Scheme 2, and the specific structures of the individual •OOQOOH H−migration pathways considered here are further summarized in Table 2.

It should be noted that only 13 representative •OOQOOH intramolecular H−migration channels are characterized in this study, rather than enumerating all conceivable reaction pathways. These target reactions are carefully selected from the kinetically favored radicals produced after the first O_2_ addition and primary H−migration. Pathways originating from high−energy conformers or featuring substantially higher energy barriers are excluded because such reactions proceed at negligible rates under typical low-temperature oxidation conditions and barely contribute to fuel consumption and chain-branching processes. Therefore, the selected 13 channels cover the most kinetically competitive H−migration pathways, including dominant 1,5 H−migration reactions and several important 1,6 H−migration channels, which can sufficiently support the development and optimization of subclass-resolved rate rules for alkylcyclohexane oxidation.

**Figure 1:**
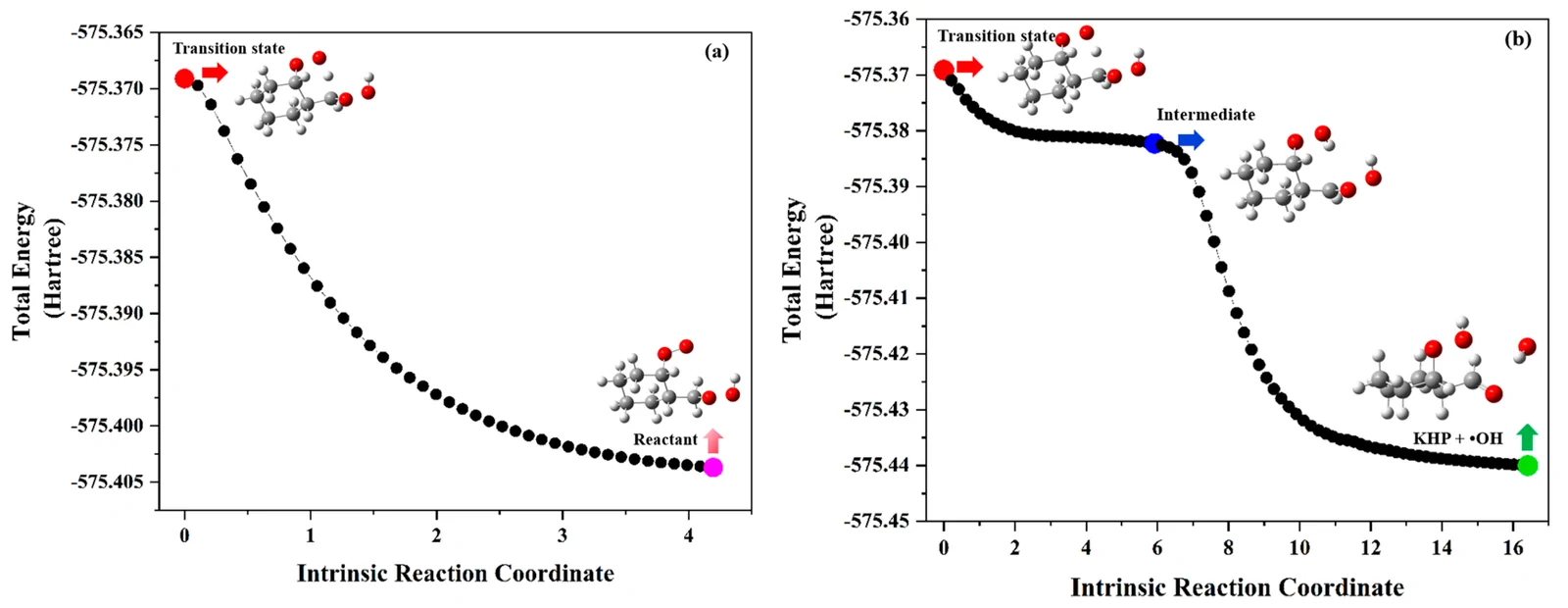
Intrinsic reaction coordinate (IRC) profiles for a representative •OOQOOH H−migration reaction: (**a**) reactant-side trajectory connecting the transition state to the corresponding •OOQOOH reactant; (**b**) product-side trajectory showing the two-stage structural evolution from the transition state through a metastable intermediate to the final KHP + •OH products. Gray, white, and red spheres represent C, H, and O atoms, respectively.

As illustrated in Scheme 2, the present work focuses on the intramolecular H−migration of •OOQOOH radicals formed after the second O_2_ addition, followed by decomposition to KHP + •OH. A representative •OOQOOH H−migration pathway was therefore selected for bidirectional intrinsic reaction coordinate (IRC) analysis [34]. As shown in Figure 1a, the reactant−side IRC trajectory connects the transition state to the corresponding •OOQOOH reactant, confirming that the located transition state is associated with the intended H−migration pathway. The product−side IRC trajectory shown in Figure 1b reveals two successive stages of structural evolution following the transition state. In the first stage, the system undergoes gradual structural reorganization and approaches a metastable intermediate at an IRC value of approximately 6. In the second stage, further bond cleavage and skeletal rearrangement occur, accompanied by a pronounced decrease in energy, ultimately leading to the formation of the thermodynamically favored ketohydroperoxide (KHP) and hydroxyl radical (•OH) products. Thus, the bidirectional IRC calculations confirm the connectivity between the •OOQOOH reactant, the H−migration transition state, and the product-side structures, while revealing the two-stage evolution toward the final products.

To further characterize the electronic−structure features associated with the H−migration process, QTAIM analysis is performed for the transition state of reaction R1. The identified bond critical points and corresponding topological parameters provide additional electronic-structure support for the proposed H−migration mechanism (see Supplementary Materials).

### 2.2. Energy Barriers for Subclasses

In most automated mechanism generators, generic rate rules are conventionally adopted [35], in which identical rate parameters (*A*, *n*, *E*) are assigned to all reactions within the same reaction class. To mitigate the uncertainties originating from this conventional lumped treatment, the intramolecular H−migration reactions of •OOQOOH radicals are further subdivided into refined subclasses in the present work, with independent rate-rule parameters established for each subclass. To quantitatively evaluate the reliability of this subclass-resolved strategy, the maximum energy barrier deviation within each category is statistically quantified. All 13 collected H−migration channels (R1−R13) are systematically classified according to three structural descriptors: the degree of substitution at the peroxyl radical-bearing carbon, the nature of the hydrogen-donor carbon, and the size of the cyclic transition state, and their corresponding energy barriers are summarized in Table 2.

Table 2 presents the energy barriers of 13 intramolecular H−migration reactions for •OOQOOH radicals in alkylcyclohexane systems, categorized into six subclasses based on the ring size of the cyclic transition state, the carbon type bearing the −OO moiety, and the carbon type donating the migrating hydrogen. The maximum energy barrier deviation within each subclass is provided to evaluate the reliability of subclass−specific rate rules. Overall, the calculated deviations within each subclass remain below 8.1 kJ mol^−1^, indicating that the refined classification strategy adopted in this work substantially improves the predictive accuracy of rate rules over the conventional lumped approach. Reactions involving H−migration from secondary C−H bonds exhibit lower energy barriers than those from primary C-H bonds. Likewise, 1,6 H−migration pathways proceeding through seven-membered cyclic transition states show considerably lower barriers than their 1,5 H−migration counterparts.

It is noteworthy that several reaction subclasses, namely 1,5−H−(t)(p), 1,6−H−(t)(p), and 1,6−H−(t)(s), each contain only one representative reaction. For these sparsely sampled subclasses, the rate rules derived herein rely exclusively on representative configurations and should thus be treated as preliminary estimates. Additional calculations for more isomers within these groups are required in future studies to enhance the generality of the obtained kinetic parameters. These single-sample subclasses are nevertheless preserved in the current classification framework: first, to establish a comprehensive scheme describing •OOQOOH H−migration reaction channels, and second, to offer benchmark references for further broader investigations.

Table 3 lists energy barriers for intramolecular H−migration of •OOQOOH radicals in alkylcyclohexanes and their matching acyclic alkanes. The stiff cyclohexane ring generates two counteracting influences, which together govern barrier differences between cyclic and noncyclic systems. First, conformational preorganization acts to reduce reaction barriers. The cyclic backbone restricts rotation of alkyl side chains, locking molecules into geometries that resemble the cyclic transition state required for hydrogen migration. By contrast, flexible acyclic alkanes need extensive C−H and C−C rotation to fold the carbon skeleton and position the peroxyl radical near the target hydrogen atom. Alkylcyclohexanes demand less structural rearrangement energy; this stabilizes the transition state and lowers the energy barrier. For most reaction channels (R1−R8, R11−R13), this beneficial effect prevails, yielding lower barriers than equivalent reactions in acyclic alkanes. Secondly, increased energy barriers may be induced by adverse steric and torsional strain. Where the peroxyl radical is bound directly to a tertiary carbon atom of the cyclohexane ring (R9 and R10), relaxation of the alkyl side chain is heavily restricted by the rigid cyclic framework. Marked steric repulsion and torsional stress are generated when the alkyl backbone is folded to construct a compact six−membered transition state. While low-strain geometries can be readily accessed by acyclic analogs, this strain cannot be alleviated due to geometric constraints originating from the cyclohexane ring. Destabilization of the transition state is therefore brought about, which raises the reaction energy barrier. For this reason, reversed barrier tendencies are observed for these two specific 1,5 H−migration channels when compared with their acyclic equivalents.

No such barrier inversion is found for the 1,6 H−migration channels R12 and R13. More space is provided by the seven−membered cyclic transition state to accommodate structural distortion and relieve steric repulsion. In addition, under comparable carbon skeleton environments, lower energy barriers are measured for secondary hydrogen migration relative to primary hydrogen migration. This trend is driven by the weaker bond dissociation energy associated with secondary C−H bonds. Overall, the cyclohexane ring generally facilitates intramolecular hydrogen migration of •OOQOOH radicals and accelerates low-temperature chain-branching pathways producing KHP + •OH, except for the special cases of ring-bound tertiary peroxyl radicals undergoing 1,5 H−migration.

### 2.3. High−Pressure−Limit Rate Constants and Rate Rules

In this work, the computed high−pressure−limit rate constants spanning 500–1500 K are fitted to the modified Arrhenius equation:

$$k\left(T\right)=AT^{n}exp\left[-\frac{E_{a}}{RT}\right]$$

The optimized fitted parameters (*A*, *n*, *E*) for each individual reaction are summarized in Table 4. For reactions belonging to the same subclass, the average rate constant at a given temperature T is calculated as:

$$k_{avg}(T)=\frac{1}{N}\sum_{i=1}^{N}k_{i}(T)$$

where *N* denotes the total number of reactions within the subclass, $k_{i}$*(T)* is the rate constant of the *i*-th reaction at temperature *T*, and $k_{avg}(T)$ stands for the corresponding average rate constant. These temperature−dependent average rate constants are subsequently refitted to the modified Arrhenius form to derive the lumped three-parameter set (*A*, *n*, *E*), which defines the subclass-specific rate rule.

**Figure 2:**
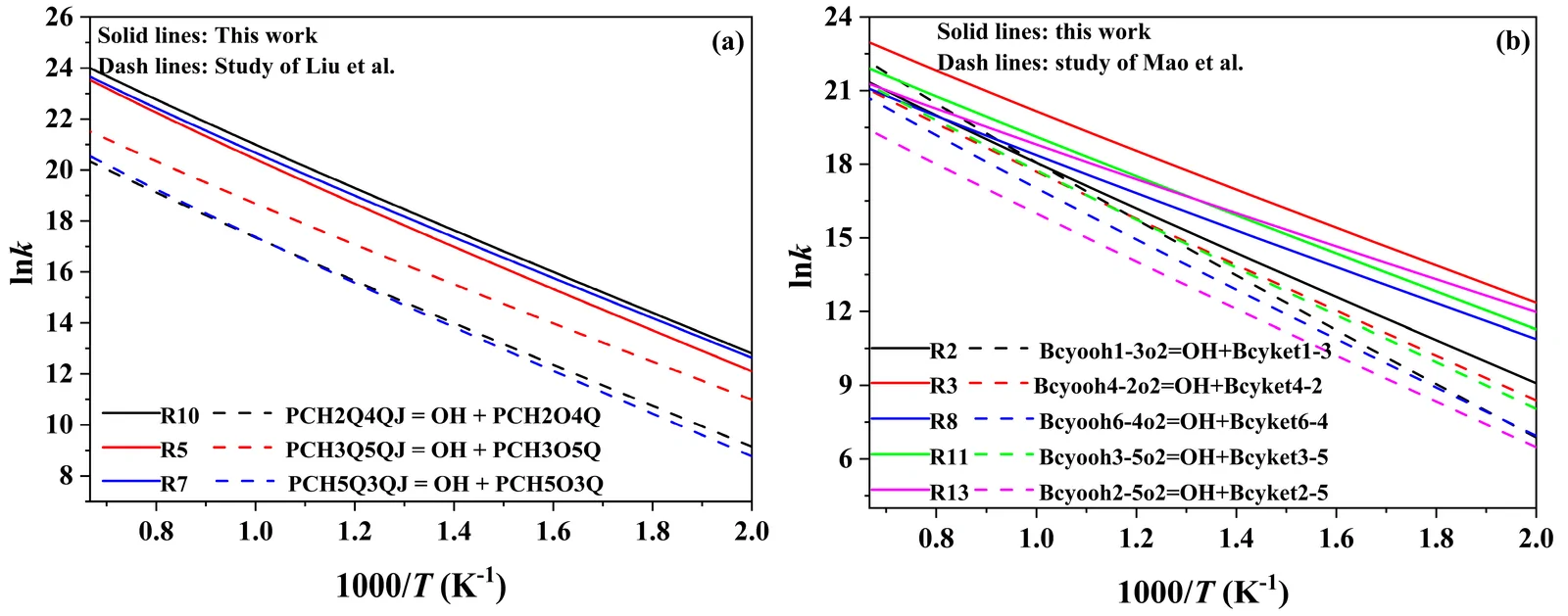
(**a**) Comparison of the high−pressure−limit rate constants of reactions R10, R5, and R7 in this study with the corresponding reactions PCH2Q4QJ = OH + PCH2O4Q, PCH3Q5QJ = OH + PCH3O5Q, and PCH5Q3QJ = OH + PCH5O3Q, respectively, from the mechanism for n-propyl cyclohexane developed by Liu et al. [9]. (**b**) Comparison of the high−pressure−limit rate constants for reactions R2, R3, R8, R11, and R13 in this study with the corresponding reactions Bcyooh1−3o2 = OH + Bcyket1−3, Bcyooh4−2o2 = OH + Bcyket4−2, Bcyooh6−4o2 = OH + Bcyket6−4, Bcyooh3−5o2 = OH + Bcyket3−5, and Bcyooh2−5o2 = OH + Bcyket2−5 in the mechanism for n-butyl cyclohexane developed by Mao et al. [11].

Direct experimental rate constants for the specific •OOQOOH intramolecular H−migration reactions investigated here are currently unavailable. Because these reactive radical intermediates are short-lived, the rate constants of individual elementary channels are difficult to resolve directly in combustion experiments. Therefore, direct experimental validation of the calculated rate constants is not presently possible. In the absence of such experimental data, the calculated results are compared with the corresponding kinetic parameters adopted in previously published alkylcyclohexane mechanisms, as shown in Figure 2.

Figure 2 compares the high−pressure−limit rate constants calculated in the present work with those adopted in previously published kinetic mechanisms for n−propylcyclohexane and n−butylcyclohexane. In Figure 2a, the calculated rate constants for R10, R5, and R7 are compared with the corresponding reactions adopted by Liu et al. [9], whereas Figure 2b compares R2, R3, R8, R11, and R13 with the corresponding rate expressions used by Mao et al. [11]. In both panels, the rate constants obtained from the present dedicated quantum chemical calculations are consistently higher than those estimated by analogy over the entire temperature range considered. Although the magnitude of the discrepancy varies among the individual reaction channels, appreciable differences are observed, particularly in the lower-temperature region. These results indicate that kinetic parameters transferred from analogous acyclic or related reaction systems may not adequately represent •OOQOOH H−migration kinetics in alkylcyclohexanes. The cyclic molecular framework imposes distinct conformational and energetic constraints that are not fully captured by simple analogy−based estimates. Therefore, dedicated quantum chemical calculations are important for developing system-specific kinetic parameters for alkylcyclohexane •OOQOOH H−migration reactions and for reducing the uncertainty associated with directly transferring rate rules from acyclic analogs.

For every investigated reaction channel, the literature rate constants remain uniformly smaller than those calculated in this work over the entire temperature window. Such noticeable discrepancies demonstrate that kinetic parameters directly transferred from alkane systems fail to properly represent •OOQOOH H−migration reactions occurring in cyclic alkylcyclohexane radicals. Unlike linear alkanes, the cyclohexane framework restricts conformational flexibility and can alter the structural rearrangement required to reach the transition state. These effects contribute to the differences in activation enthalpies and calculated rate constants between alkylcyclohexanes and their linear alkane analogs. This observation further demonstrates the necessity of computing dedicated kinetic data for alkylcyclohexane species, as adopted alkane parameters would bring systematic deviations in simulating low-temperature chain-branching processes.

## 3. Methods

Open-shell doublet species, including the •OOQOOH reactants, H−migration transition states, and •OH radicals, are treated using the unrestricted Kohn–Sham formalism (UB3LYP) [36], whereas the closed−shell singlet ketohydroperoxide (KHP) products are treated using the restricted Kohn–Sham formalism (RB3LYP). The calculated <*S*^2^> values are examined to evaluate the extent of spin contamination, and the corresponding results are summarized in Table S2 of the Supplementary Materials.

All reactants and products possess solely real vibrational frequencies, whereas each transition state exhibits exactly one imaginary frequency. The vibrational mode corresponding to this imaginary frequency connects reactants and products along the reaction coordinate. The corresponding thermodynamic correction data are provided in Table S4 of the Supplementary Materials. All aforementioned calculations are implemented using the Gaussian 16 Revision C.01 quantum chemistry package (Gaussian, Inc., Wallingford, CT, USA) [37]. Single-point energies of these optimized geometries are further evaluated via the CBS-QB3 composite method [36]. The activation enthalpies reported in Table 2 and Table 3 are calculated at 298.15 K as ${\Delta H}_{298}^{\neq }$ = *H*_TS,298_ − *H*_R,298_, where *H*_TS,298_ and *H*_R,298_ denote the enthalpies of the transition state and the corresponding reactant, respectively. The CBS-QB3 enthalpies, including thermal corrections at 298.15 K, are used for this purpose, and the resulting activation enthalpies are reported in kJ mol^−1^. Optimized geometries of all species, including reactants, transition states, and products, are provided in the Supplementary Materials.

Based on the calculated potential energy surfaces, conventional transition state theory (TST) [38] is first utilized to compute the high−pressure−limit rate constants (*k*, s^−1^) for the investigated reactions. Quantum tunneling effects cannot be neglected for elementary reactions at low temperatures. The Eckart model [39] is applied to evaluate the tunneling correction factor denoted as *κ*(*T*), which quantifies the influence of tunneling on rate constants. In this work, ChemRate program version 1.5.8 (National Institute of Standards and Technology: Gaithersburg, MD, USA, 2009), established by Mokrushin and Tsang [40], is employed to obtain high−pressure−limit rate constants of all investigated reactions within the temperature range of 500−1500 K.

The harmonic oscillator approximation is employed to evaluate vibrational partition functions. Nevertheless, this approximation yields considerable errors for low-frequency internal rotations associated with alkyl side chains, −OO and −QOOH branches. These internal rotational motions are therefore treated as one-dimensional hindered rotors [41]. Using the same level of theory as that employed for geometry optimization, relaxed torsional potential-energy scans over 360° with a 10° increment are performed for each relevant rotatable single bond. These scans are used not only to construct the hindrance potential functions for the one-dimensional (1−D) hindered-rotor treatment but also to explore the conformational space of the investigated species. Low-energy minima identified from the torsional potential-energy profiles were examined and fully optimized, and the lowest-energy reactant conformer identified through this procedure is subsequently used for transition-state searches and reaction-path calculations. A detailed conformational analysis of the representative R5 reaction is provided in the Supplementary Materials.

## 4. Conclusions

Quantum chemical kinetic investigations on critical •OOQOOH intramolecular H−migration reactions during low-temperature oxidation of alkylcyclohexanes have been carried out, and the main conclusions are summarized as follows:

(1) High−pressure−limit rate constants of 13 representative intramolecular H−migration pathways of •OOQOOH are obtained. These reactions are categorized into six subclasses according to the ring size of the cyclic transition state, the carbon type bearing the –OO moiety, and the carbon type donating the migrating hydrogen. Modified Arrhenius parameters for individual reactions and subclass-averaged rate rules are fitted. This structural classification strategy groups analogous H−migration reactions and provides a basis for developing rate rules in automated mechanism generation.
(2) The cyclohexane ring exerts competing effects on the activation barriers compared with acyclic alkane analogs. The cyclic framework restricts alkyl chain rotation and promotes conformational preorganization, which lowers the barriers for most H−migration pathways. However, for 1,5-H−migration reactions involving ring-attached tertiary peroxyl radicals, steric and torsional strain introduced by geometric confinement destabilizes the transition states and leads to higher barriers than the corresponding acyclic reactions. Such barrier inversion is not observed for the investigated 1,6 H−migration pathways, likely due to the greater spatial flexibility of the seven-membered transition states.
(3) Comparison with kinetic parameters adopted in existing alkylcyclohexane mechanisms shows that rate constants estimated from linear alkane analogs are consistently lower than the present calculated values over the investigated temperature range. The differences originate from the combined effects of conformational preorganization and steric constraints imposed by the cyclohexane ring. Therefore, direct transfer of kinetic parameters from acyclic systems may introduce significant uncertainties into low-temperature oxidation models of alkylcyclohexanes.
(4) Unlike previous mechanisms that relied mainly on analogy-based estimation, this work addresses the lack of dedicated kinetic data for •OOQOOH H−migration reactions in alkylcyclohexanes by providing ring-structure-specific quantum chemical calculations. The resulting kinetic parameters offer a more reliable basis for refining low-temperature oxidation mechanisms of cyclohexane fuels and reducing uncertainties associated with analogy-based rate estimation. Future work will focus on expanding the reaction dataset within each subclass and performing ignition simulations to further evaluate the impact of the revised kinetic parameters.

## Figures and Tables

**Table 1:** Electronic energies at 0 K (kJ mol^−1^) of equatorial and axial conformers for methylcyclohexane and ethylcyclohexane.

Molecular System	Conformational No.	Spatial Orientation of Substituent	Electronic Energy
Methylcyclohexane	MCH ^a^-e ^c^	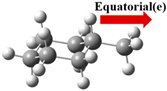	−720,914.5
Methylcyclohexane	MCH-a ^d^	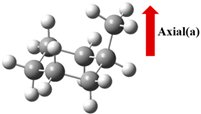	−720,906.5
Ethylcyclohexane	ECH ^b^-e	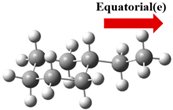	−823,899.9
Ethylcyclohexane	ECH-a	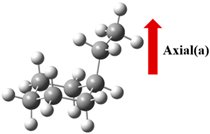	−823,891.9

^a^ MCH = methylcyclohexane. ^b^ ECH = ethylcyclohexane. ^c^ The suffix “e”means the alkyl substituent is located at the equatorial position of chair cyclohexane. ^d^ The suffix “a” means the alkyl substituent is located at the axial position of chair cyclohexane.

**Table 2:** Activation enthalpies (${\Delta H}_{298}^{\neq }$, kJ mol^−1^) for the H−migration reactions of •OOQOOH yielding KHP + •OH.

No.	Reactions	Activation Enthalpy, ${\Delta H}_{298}^{\neq }$
R1	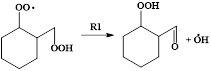	70.4
R2	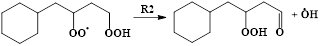	78.5
**1,5 ^a^-H-(s) ^b^(p) ^c^**		(74.5 ^d^, 8.1 ^e^)
R3	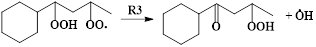	68.2
R4	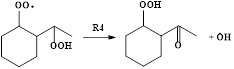	63.1
R5	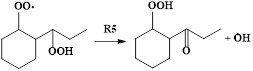	66.1
R6	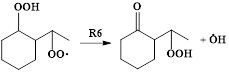	68.0
R7	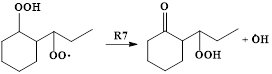	70.0
R8	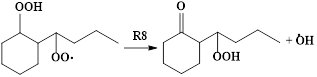	70.1
**1,5-H-(s)(s)**		(67.6, 7.0)
R9	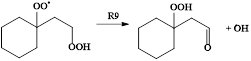	82.4
**1,5-H-(t)(p)**		(82.4, 0)
R10	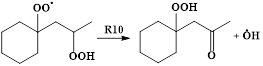	71.9
R11	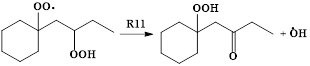	71.7
**1,5-H-(t)(s)**		(71.8, 0.2)
R12	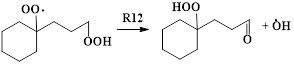	63.0
**1,6-H-(t)(p)**		(63.0, 0)
R13	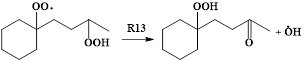	61.9
**1,6-H-(t)(s)**		(61.9, 0)

^a^ Denotes the type of intramolecular H−migration, where 1,5− and 1,6− represent six-membered and seven−membered cyclic transition states, respectively. ^b^ Indicates the carbon type bonded to the peroxyl radical (•OOQOOH), with s and t denoting secondary and tertiary carbon atoms, respectively. ^c^ Represents the carbon type that provides the migrated hydrogen atom, where p and s correspond to primary and secondary carbon atoms, respectively. ^d^ The average energy barrier of reactions in a subclass. ^e^ The maximum deviation of energy barriers in a subclass.

**Table 3:** Activation enthalpies (${\Delta H}_{298}^{\neq }$, kJ mol^−1^) for 1,5 H−migration reactions of •OOQOOH radicals in alkylcyclohexane and corresponding alkane systems.

	Alkyl Cyclohexane Reactions	${\Delta H}_{298}^{\neq }$	Alkane Reactions	${\Delta H}_{298}^{\neq }$
**1,5-H-(s)(p)**	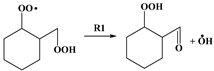	70.4	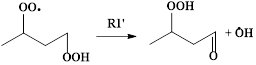	81.1
	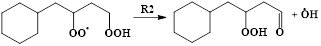	78.5	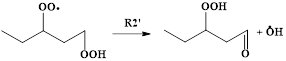	81.6
**1,5-H-(s)(s)**	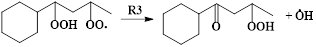	68.2	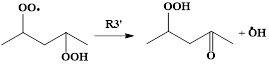	73.7
	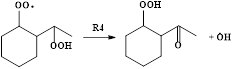	63.1	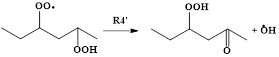	74.3
	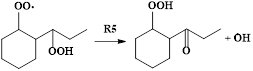	66.1	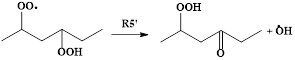	81.1
	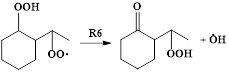	68.0	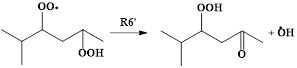	75.2
	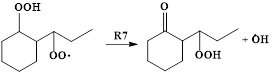	70.0	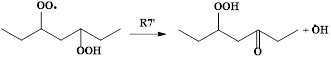	81.6
	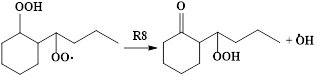	70.1	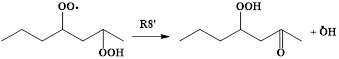	74.5
**1,5-H-(t)(p)**	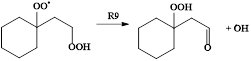	82.4	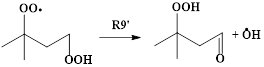	70.6
**1,5-H-(t)(s)**	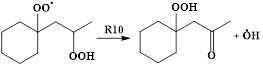	71.9	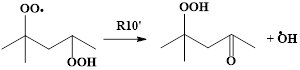	68.3
	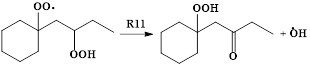	71.7	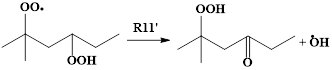	75.8
**1,6-H-(t)(p)**	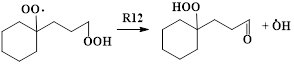	63.0	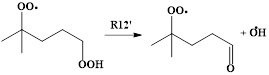	73.0
**1,6-H-(t)(s)**	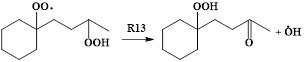	61.9	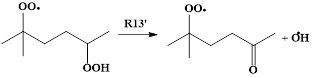	62.2

**Table 4:** Calculated rate constants and derived rate rules in each subclass for the reactions with KHP + OH• product at high pressure.

	Modified Arrhenius Parameters		
Reaction	*A* (s^−1^)	*n*	*E* (cal mol^−1^)
R1	2.66 × 10^6^	1.8	13,380.1
R2	2.43 × 10^6^	1.6	15,596.4
**1,5-H-(s)(p)**	**2.32 × 10^7^**	**1.6**	**15,630.1**
R3	1.29 × 10^8^	1.2	13,810.3
R4	6.33 × 10^6^	1.6	12,001.2
R5	1.10 × 10^6^	2.0	13,839.7
R6	1.96 × 10^8^	1.2	13,815.8
R7	5.27 × 10^6^	1.7	13,579.3
R8	1.91 × 10^7^	1.2	13,232.4
**1,5-H-(s)(s)**	**1.44 × 10^6^**	**1.8**	**12,751.4**
R9	4.56 × 10^6^	1.8	16,275.9
**1,5-H-(t)(p)**	**4.56 × 10^6^**	**1.8**	**16,275.9**
R10	3.48 × 10^7^	1.6	14,134.9
R11	4.03 × 10^8^	0.9	14,296.3
**1,5-H-(t)(s)**	**3.18 × 10^7^**	**1.5**	**14,126.4**
R12	1.65 × 10^7^	1.2	12,214.7
**1,6-H-(t)(p)**	**1.65 × 10^7^**	**1.2**	**12,214.7**
R13	3.19 × 10^7^	1.1	12,055.9
**1,6-H-(t)(s)**	**3.19 × 10^7^**	**1.1**	**12,055.9**

## Data Availability

The original contributions presented in this study are included in the article/Supplementary Material. Further inquiries can be directed to the corresponding authors.

## Supplementary Materials

The following supporting information can be downloaded at: https://www.mdpi.com/article/10.3390/molecules31183299/s1.

## References

[1] Shen Y.H., Tian Z.M., Li W., Ji Y.X., Yan Y.W. (2025). Theoretical Study of the Effect of Conformational Structures on the Secondary Oxidation Reactions of cis-1,3-Dimethylcyclohexane. Chem. J. Chin. Univ..

[2] Westbrook C.K., Smith P.J. (2006). Basic Research Needs for Clean and Efficient Combustion of 21st Century Transportation Fuels.

[3] Sarathy S.M., Farooq A., Kalghatgi G.T. (2018). Recent progress in gasoline surrogate fuels. Prog. Energy Combust. Sci..

[4] Edwards T., Maurice L.Q. (2001). Surrogate Mixtures to Represent Complex Aviation and Rocket Fuels. J. Propul. Power.

[5] Pitz W.J., Mueller C.J. (2011). Recent Progress in the Development of Diesel Surrogate Fuels. Prog. Energy Combust. Sci..

[6] Liu G., Yan B., Chen G. (2013). Technical review on jet fuel production. Renew. Sustain. Energy Rev..

[7] Balster L.M., Corporan E., DeWitt M.J., Edwards J.T., Ervin J.S., Graham J.L., Lee S.Y., Pal S., Phelps D.K., Rudnick L.R. (2008). Development of an advanced, thermally stable, coal-based jet fuel. Fuel Process. Technol..

[8] Zhang X., Yan H., Zhu L.J., Li T., Wang S.R. (2020). Hydrodeoxygenation of Lignin-Derived Monomers and Dimers over a Ru Supported Solid Super Acid Catalyst for Cycloalkane Production. Adv. Sustain. Syst..

[9] Liu M.X., Fang R.Z., Sung C.J., Aljohani K., Farooq A., Almarzooq Y., Mathieu O., Petersen E.L., Dagaut P., Zhao J. (2022). A comprehensive experimental and modeling study of n-propylcyclohexane oxidation. Combust. Flame.

[10] Mao Y.B., Wang S.X., Wu Z.Y., Qiu Y., Yu L., Ruan C., Chen F., Zhu L., Lu X.C. (2019). An experimental and kinetic modeling study of n-butylcyclohexane over low-to-high temperature ranges. Combust. Flame.

[11] Mao Y.B., Li A., Zhu L., Wu Z.Y., Yu L., Wang S.X., Raza M., Lu X.X. (2019). A detailed chemical mechanism for low to high temperature oxidation of n-butylcyclohexane and its validation. Combust. Flame.

[12] Sirjean B., Buda F., Hakka H., Glaude P.A., Fournet R., Warth V., Battin-Leclerc F., Ruiz-Lopez M. (2007). The autoignition of cyclopentane and cyclohexane in a shock tube. Proc. Combust. Inst..

[13] Yao X.X., Wang J.B., Yao Q., Li Y.Q., Li Z.R., Li X.Y. (2019). Pressure-dependent rate rules for intramolecular H−migration reactions of normal-alkyl cyclohexylperoxy radicals. Combust. Flame.

[14] Zador J., Taatjes C.A., Fernandes R.X. (2011). Kinetics of elementary reactions in low-temperature autoignition chemistry. Prog. Energy Combust. Sci..

[15] Liu M.X., Hui X., Xue X., Lin Y.Z., Zhou C.W. (2023). From electronic structure to model application for alkyl cyclohexane combustion chemistry: H-atom abstraction reactions by HO_2_ radical. Phys. Chem. Chem. Phys..

[16] Yang M., Wang J. (2023). Multi-structural variational kinetics study on hydrogen abstraction reactions of cyclopentanol and cyclopentane by hydroperoxyl radical with anharmonicity, recrossing and tunneling effects. Phys. Chem. Chem. Phys..

[17] Sharma S., Raman S., Green W.H. (2010). Intramolecular hydrogen migration in alkylperoxy and hydroperoxyalkylperoxy radicals: Accurate treatment of hindered rotors. J. Phys. Chem. A.

[18] Miyoshi A. (2011). Systematic computational study on the unimolecular reactions of alkylperoxy (RO_2_), hydroperoxyalkyl (QOOH), and hydroperoxyalkylperoxy (O_2_QOOH) radicals. J. Phys. Chem. A.

[19] Zhang F., Dibble T.S. (2011). Effects of olefin group and its position on the kinetics for intramolecular H-shift and HO_2_ elimination of alkenylperoxy radicals. J. Phys. Chem. A.

[20] Li S.J., Tan N.X., Yao Q., Li Z.R., Li X.Y. (2015). Calculation of rate constants for intramolecular hydrogen migration reactions of alkylperoxy radicals. Acta Phys.-Chim. Sin..

[21] Villano S.M., Huynh L.K., Carstensen H.H. (2011). High-pressure rate rules for alkyl + O_2_ reactions. 1. The dissociation, concerted elimination, and isomerization channels of the alkyl peroxy radical. J. Phys. Chem. A.

[22] Yang Y., Boehman A.L., Simmie J.M. (2010). Uniqueness in the low temperature oxidation of cycloalkanes. Combust. Flame.

[23] Yao X.X., Li J.Q., Li Z.R. (2025). Computational Kinetic Study on the Intramolecular H−Migration of Hydroperoxyalkylperoxy Radicals (•OOQOOH) in Normal-Alkyl Cyclohexanes. Molecules.

[24] Yao Q., Sun X.H., Li Z.R., Chen F.F., Li X.Y. (2017). Pressure-dependent rate rules for intramolecular H−migration reactions of hydroperoxyalkylperoxy radicals in low temperature. J. Phys. Chem. A.

[25] Yang Y., Boehman A.L., Simmie J.M. (2010). Effects of Molecular Structure on Oxidation Reactivity of Cyclic Hydrocarbons: Experimental Observations and Conformational Analysis. Combust. Flame.

[26] Freeman F., Tsegai Z.M., Kasner M.L., Hehre W.J. (2000). A Comparison of the ab Initio Calculated and Experimental Conformational Energies of Alkylcyclohexanes. J. Chem. Educ..

[27] Knepp A.M., Meloni G., Jusinski L.E., Taatjes C.A., Cavallotti C., Klippenstein S.J. (2007). Measurements theory, and modeling of OH and HO2 formation in the reaction of cyclohexyl radicals with O2. Phys. Chem. Chem. Phys..

[28] Xing L.L., Zhang F., Zhang L.D. (2015). Theoretical studies for reaction kinetics of cyC6H11CH2 radical with O_2_. Proc. Combust. Inst..

[29] Xing L.L., Zhang L.D., Zhang F., Jiang J. (2017). Theoretical kinetic studies for low temperature oxidation of two typical methylcyclohexyl radicals. Combust. Flame.

[30] Serinyel Z., Herbinet O., Frottier O., Dirrenberger P., Warth V., Glaude P.A., Battin-Leclerc F. (2013). An experimental and modeling study of the low- and high-temperature oxidation of cyclohexane. Combust. Flame.

[31] Weber B.W., Pitz W.J., Mehl M., Silke E.J., Davis A.C., Sung C.J. (2014). Experiments and modeling of the autoignition of methylcyclohexane at high pressure. Combust. Flame.

[32] Bissoonauth T., Wang Z.D., Mohamed S.Y., Wang J.Y., Chen B.J., Rodriguez A., Frottier O., Zhang X.Y., Zhang Y., Cao C.C. (2019). Methylcyclohexane pyrolysis and oxidation in a jet-stirred reactor. Proc. Combust. Inst..

[33] Zou J.B., Li Y.Y., Ye L.L., Jin H.F. (2022). A comprehensive study on low-temperature oxidation chemistry of cyclohexane. I. Conformational analysis and theoretical study of first and second oxygen addition. Combust. Flame.

[34] Gonzalez C., Schlegel H.B. (1989). An improved algorithm for reaction path following. J. Chem. Phys..

[35] Li Y.Q., Yao X.X., Sun X.H., Li Z.R., Li X.Y. (2020). Automatic construction of transition states and on-the-fly accurate kinetic calculations for reaction classes in automated mechanism generators. Comput. Theor. Chem..

[36] Montgomery J.A., Frisch M.J., Ochterski J.W., Petersson G.A. (1999). A complete basis set model chemistry. VI. Use of density functional geometries and frequencies. J. Chem. Phys..

[37] Frisch M.J., Trucks G.W., Schlegel H.B., Scuseria G.E., Robb M.A., Cheeseman J.R., Scalmani G., Barone V., Petersson G.A., Nakatsuji H. (2016). Gaussian 16.

[38] Truhlar D.G., Garrett B.C., Klippenstein S.J. (1996). Current Status of Transition-State Theory. J. Phys. Chem. A.

[39] Johnston H.S., Heicklen J. (1962). Tunnelling corrections for unsymmetrical eckart potential energy barriers. J. Phys. Chem..

[40] Mokrushin V., Tsang W. (2009). Chemrate.

[41] Pitzer K.S., Gwinn W.D. (1942). Energy levels and thermodynamic functions for molecules with internal rotation I. Rigid frame with attached tops. J. Chem. Phys..

