# Supplementary material for: Intramolecular H−Migration Kinetics of •OOQOOH Radicals for KHP Formation During Low-Temperature Oxidation of Alkylcyclohexanes
Source: Molecules. 2026 Sep 17;31(18):3299. doi: 10.3390/molecules31183299 (PMC13609893; doi:10.3390/molecules31183299)
Supplement: Supplementary file 1 [file molecules-31-03299-s001.zip › molecules-4489797-supplementary.pdf]

Intramolecular H-Migration Kinetics of •OOQOOH Radicals for KHP Formation during Low-Temperature Oxidation of Alkylcyclohexanes

Xiaoxia Yao[1],Yuheng Liu[1], Ying Xuan[1], Junjiang Guo[2], Mingxia Liu[3], Zerong Li[4*], Wenjiong Hai[1*]

[1]*Aviation Maintenance Industry College, Chengdu Aeronautic Polytechnic University, Chengdu 610100, China*

[2]*School of Chemical Engineering, Guizhou Institute of Technology, Guiyang 550003, China*

[3]*School of Physics and New Energy, Chongqing University of Technology, Chongqing 401135, China*

[4]*College of Chemistry, Sichuan University, Chengdu 610064, China*

(1) Potential energy profiles for internal rotations
(2) The spin eigenvalues and the S^2 computed by Gaussian16 package for the species involved in this study.
(3) QTAIM analysis of the representative R5 H-migration transition state
(4) Thermodynamic properties and vibrational characteristics of reactants, transition states, and products
(5) Cartesian coordinates for all reactants, transition states and products

(1) Potential energy profiles for internal rotations

In the present study, one-dimensional (1-D) hindered internal rotors are used to treat the low-frequency vibrational modes associated with torsional motions about single bonds in the reactants, transition states, and products. The reactant of R5 is selected as a representative example, and the relaxed potential-energy profiles for internal rotations about the relevant C–C, C–O, and O–O bonds are shown in Fig. S1. For each relevant rotatable single bond, a 360° relaxed torsional scan with a 10° increment was performed. Low-energy minima identified from the torsional scans are subsequently examined and fully optimized, and the lowest-energy conformer identified from this procedure is used as the reference reactant structure for the transition-state search and reaction-path calculations.

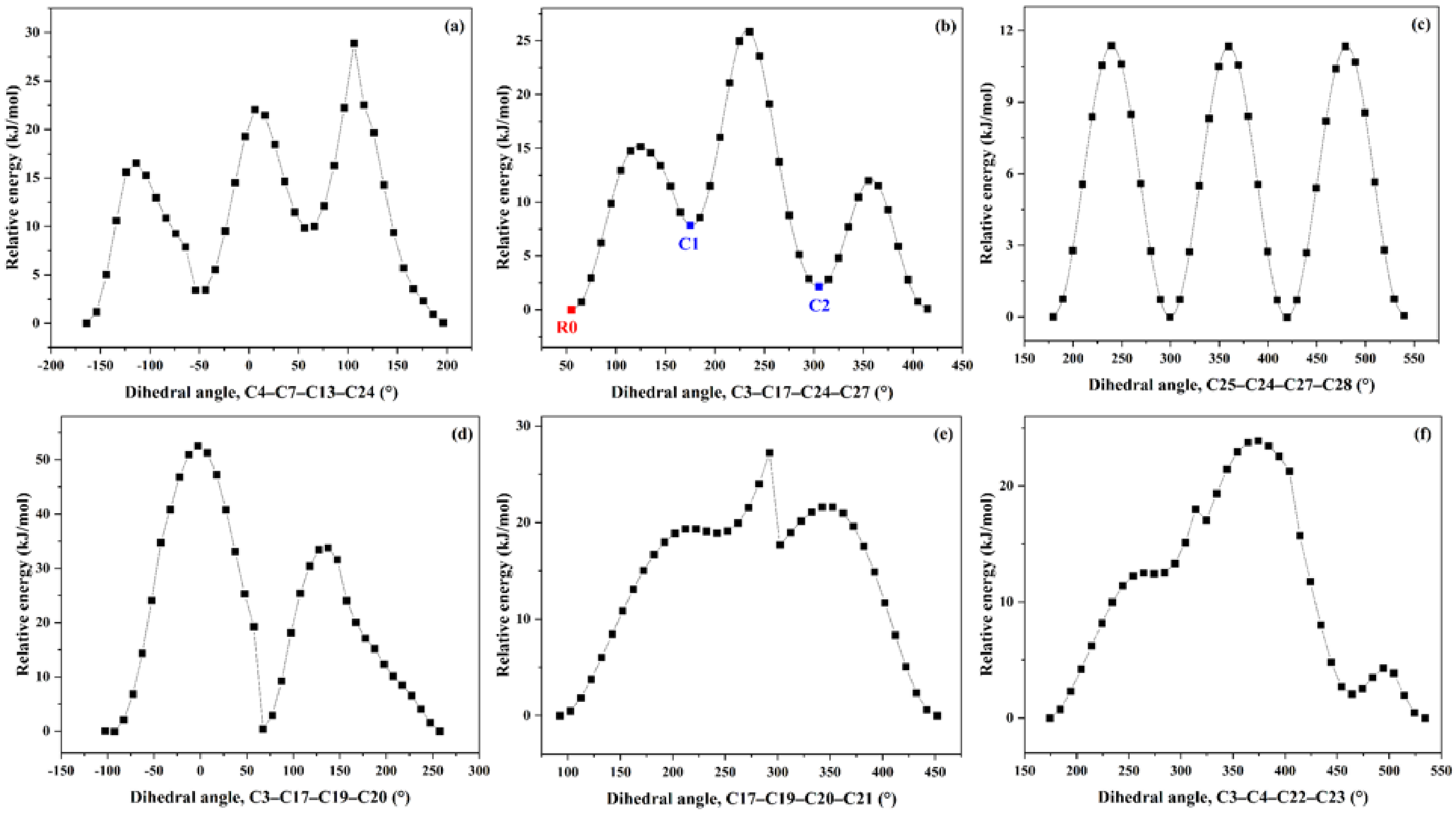

Figure S1. Relaxed torsional potential-energy profiles for internal rotations in the R5 reactant calculated at the B3LYP/CBSB7 level. The energies in each panel are referenced to the minimum energy along the corresponding torsional scan.

To further evaluate the influence of conformational flexibility on the calculated reaction barrier, two additional low-energy conformers of the R5 reactant, C1 and C2, are selected from the local minima identified in the torsional potential-energy profile shown in Fig. S1(b). Both conformers are fully optimized, and the corresponding transition states are subsequently located and verified. The relative conformational energies and reaction barriers are summarized in Table S1. Here, R0 denotes the lowest-energy reactant conformer employed in the original calculations; ΔE_{conf} represents the energy of each conformer relative to R0; $E_{TS}-E_{conf}$ denotes the conformer-specific reaction barrier; and E_{TS}-E_{R0} represents the energy of the corresponding transition state referenced to the common lowestg-energy reactant conformer R0.

Table S1. Relative conformational energies and reaction barriers of different R5 reactant conformers (kJ mol^{-1}).

R5 reactant conformer	ΔE_{conf} (kJ mol^{-1})	$E_{TS}-E_{conf}$ (kJ mol^{-1})	E_{TS}-E_{R0} (kJ mol^{-1})
R0	0.0	66.1	66.1
C1	5.6	60.3	65.9
C2	0.3	72.0	72.3

Although the conformer-specific barriers range from 60.3 to 72.0 kJ mol^{-1}, comparison of the transition-state energies referenced to the common lowest-energy reactant conformer R0 gives values of 66.1, 65.9, and 72.3 kJ mol^{-1} for the R0, C1, and C2 pathways, respectively. The R0 and C1 pathways are therefore essentially isoenergetic, with their transition-state energies differing by only 0.2 kJ mol^{-1}. Thus, although different low-energy conformers exhibit appreciable differences in their conformer-specific barriers, explicit consideration of these additional conformers does not appreciably change the minimum transition-state energy for the representative R5 reaction.

(2) The spin eigenvalues and the S^2 computed by Gaussian16 package for the species involved in this study.

Table S2. Calculated $<S^2>$ values and deviations from the ideal spin eigenvalues for the investigated species.

Species	Spin eigenvalues	$<S^2>$	Relative error (%)
R1	0.75	0.7538	0.5
TS1	0.75	0.7559	0.8
P1		0.000 (closed-shell singlet)	
R2	0.75	0.7536	0.5
TS2	0.75	0.7556	0.7
P2		0.000 (closed-shell singlet)	
R3	0.75	0.7535	0.5
TS3	0.75	0.7559	0.8
P3		0.000 (closed-shell singlet)	
R4	0.75	0.7539	0.5
TS4	0.75	0.7560	0.8
P4		0.000 (closed-shell singlet)	
R5	0.75	0.7538	0.5
TS5	0.75	0.7561	0.8
P5		0.000 (closed-shell singlet)	
R6	0.75	0.7536	0.5
TS6	0.75	0.7559	0.8

P6		0.000 (closed-shell singlet)	
R7	0.75	0.7536	0.5
TS7	0.75	0.7559	0.8
P7		0.000 (closed-shell singlet)	
R8	0.75	0.7536	0.5
TS8	0.75	0.7559	0.8
P8		0.000 (closed-shell singlet)	
R9	0.75	0.7540	0.5
TS9	0.75	0.7556	0.7
P9		0.000 (closed-shell singlet)	
R10	0.75	0.7540	0.5
TS10	0.75	0.7557	0.8
P10		0.000 (closed-shell singlet)	
R11	0.75	0.7540	0.5
TS11	0.75	0.7556	0.7
P11		0.000 (closed-shell singlet)	
R12	0.75	0.7539	0.5
TS12	0.75	0.7552	0.7
P12		0.000 (closed-shell singlet)	
R13	0.75	0.7538	0.5
TS13	0.75	0.7549	0.7
P13		0.000 (closed-shell singlet)	
OH	0.75	0.7517	0.2

The calculated <S^2> values for all doublet reactants,transition states and •OH deviate from the ideal value of 0.75 by less than 1%, indicating negligible spin contamination. The ketohydroperoxide (KHP) products are closed-shell singlet species with an expected <S^2> value of 0.

(3) QTAIM analysis of the representative R5 H-migration transition state

To further elucidate the electronic-structure characteristics associated with the intramolecular H-migration process, QTAIM analysis of a representative •OOQOOH H-Migration transition state (R1). As shown in Figure S2, two (3,−1) bond critical points (BCPs) are identified along the C14···H15 and H15···O23 interactions, together with the corresponding bond paths. The relevant topological parameters are summarized in Table S3. The electron densities, ρ(r), at the C14···H15 and H15···O23 BCPs are 0.144762 and 0.187752 a.u., respectively. The corresponding Laplacians, $\nabla^2\rho(\mathrm{r})$, are −0.208804 and −0.216161 a.u., while the total energy densities, H(r), are −0.092816 and −0.150914 a.u., respectively. The negative values of both $\nabla^2\rho(\mathrm{r})$ and H(r) are consistent with pronounced shared-shell character for

these interactions. The simultaneous presence of the C14···H15 and H15···O23 bond paths is consistent with the three-center configuration of the H-transfer transition state, in which the original C–H bond is being weakened while the new O–H bond is being formed. Combined with the IRC analysis presented in the main text, these QTAIM results provide additional electronic-structure support for the proposed intramolecular H-migration mechanism.

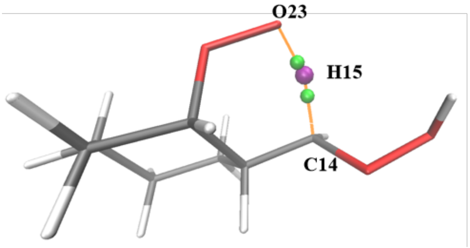

Figure S2. QTAIM molecular graph of the transition state for reaction R1. Gray, white, and red spheres represent C, H, and O atoms, respectively. The green spheres denote the (3,−1) bond critical points associated with the C14···H15 and H15···O23 interactions, and the orange lines represent the corresponding bond paths.

Table S3. QTAIM topological parameters at the bond critical points of the representative R1 H-migration transition state (a.u.).

Interation	$\rho(\mathbf{r})$	$\nabla^2\rho(\mathbf{r})$	$G(\mathbf{r})$	$V(\mathbf{r})$	$H(\mathbf{r})$
C14···H15	0.144762	-0.208804	0.040615	-0.133431	-0.092816
H15···O23	0.187752	-0.216161	0.096874	-0.247788	-0.150914

(4) Thermodynamic properties and vibrational characteristics of reactants, transition states, and products

Table S4. Thermodynamic properties and vibrational characteristics of reactants, transition states, and products involved in the •OOQOOH intramolecular H-migration reactions.

Species	ZPE correction (kJ mol⁻¹)	Thermal correction to Energy (kJ mol⁻¹)	Thermal correction to Enthalpy (kJ mol⁻¹)	Thermal correction to Gibbs Free Energy (kJ mol⁻¹)	Imaginary frequency (cm⁻¹)
R1	530.25	559.65	562.13	428.63	None
TS1	514.39	542.56	545.04	417.45	−1689.0
P1	492.16	517.26	519.73	401.57	None
R2	756.12	794.82	797.30	641.60	None
TS2	737.56	776.39	778.87	622.40	−1667.90
P2	714.33	750.04	752.52	606.12	None
R3	754.25	793.39	795.87	641.72	None
TS3	737.95	776.22	778.70	628.14	−1614.16
P3	714.55	750.62	753.10	606.79	None
R4	603.49	636.60	639.08	500.62	None
TS4	587.98	619.53	622.00	488.34	−1687.53
P4	565.32	594.65	597.14	468.50	None

R5	678.28	714.81	717.28	570.77	None
TS5	662.23	697.98	700.45	556.29	−1643.86
P5	640.56	673.07	675.55	539.76	None
R6	605.69	637.80	640.28	504.66	None
TS6	588.34	619.99	622.47	488.70	−1755.08
P6	565.22	594.53	597.01	468.76	None
R7	680.06	715.92	718.40	573.41	None
TS7	662.81	698.17	700.64	557.71	−1749.75
P7	639.70	672.72	675.19	537.56	None
R8	754.47	793.96	796.44	642.06	None
TS8	737.08	776.11	778.59	626.07	−1748.66
P8	714.39	750.97	753.45	606.62	None
R9	604.82	636.77	639.25	504.20	None
TS9	587.58	619.33	621.81	485.79	−1659.47
P9	565.17	593.71	596.19	470.96	None
R10	677.35	713.20	715.68	572.40	None
TS10	660.23	695.72	698.20	555.41	−1671.62
P10	639.08	672.21	674.69	537.46	None
R11	751.97	791.54	794.02	641.32	None
TS11	735.27	774.41	776.89	624.98	−1659.59
P11	713.83	749.60	752.07	609.10	None
R12	679.58	715.37	717.85	572.78	None
TS12	665.93	698.83	701.31	564.31	−1642.08
P12	643.20	676.30	678.78	544.54	None
R13	752.07	791.85	794.33	640.50	None
TS13	738.06	775.27	777.75	631.92	−1575.29
P13	713.92	749.65	752.13	609.06	None

(5) Cartesian coordinates for all reactants, transition states and products

R1-R

C	2.42172000	-1.05183200	-0.43829900
C	0.94258800	-1.45688100	-0.51874500
C	0.12739000	-0.85996700	0.64336200
C	0.29620000	0.66596300	0.70142700
C	1.76627100	1.08437500	0.77103500
C	2.59312700	0.47224500	-0.37015600
H	0.50884100	-1.12235000	-1.46600000
H	0.84988800	-2.54751300	-0.49885300
H	2.87202500	-1.50822400	0.45311400
H	2.96567000	-1.45106400	-1.29944500
H	-0.29447200	1.11237200	1.50229000
H	1.82568800	2.17623200	0.75760900
H	2.15786900	0.75444800	1.74064500
H	2.27623000	0.91706400	-1.31904500

H	3.64738600	0.73229100	-0.23819000
H	0.56665600	-1.22687100	1.58380200
C	-1.32907500	-1.34612900	0.66816600
H	-1.85791800	-0.98549900	1.55284400
O	-2.09103000	-0.98168900	-0.48633900
O	-3.04060500	0.04276300	-0.11371100
H	-2.62758100	0.84238000	-0.48511700
O	-0.25315300	1.24274700	-0.54438900
O	-1.17385600	2.15895900	-0.31982700
H	-1.33045400	-2.44214600	0.68972300
TS			
C	-1.92209100	0.80482500	1.03574700
C	-0.46055300	0.76412300	0.59517600
C	0.02180600	-0.67945200	0.24666800
C	-0.93980300	-1.36126600	-0.74360900
C	-2.39063600	-1.33030100	-0.24490300
C	-2.85890400	0.10185200	0.04325100
H	0.19688600	1.18990500	1.36026800
H	-0.87551600	-0.84850500	-1.70937200
H	-0.61686100	-2.39384300	-0.90840100
H	-3.04461600	-1.80013600	-0.98567800
H	-2.47249400	-1.93248900	0.66957800
H	-2.88715800	0.67162200	-0.89083300
H	-3.87785600	0.09509400	0.44177200
C	1.42829500	-0.62670300	-0.30446700
H	1.39812000	0.61065800	-0.85425900
O	2.34736800	-0.58118800	0.73122800
O	3.67531600	-0.44844400	0.16282600
H	3.84244700	0.49122500	0.33086200
H	-2.21461900	1.84854300	1.17816800
H	-1.98510000	0.31839600	2.01623700
H	0.03781100	-1.24333900	1.18991500
O	-0.35235100	1.56827700	-0.58367000
O	1.02024500	1.74696800	-0.87442400
H	1.70564900	-1.33049700	-1.09310800
P			
C	1.32315300	-1.15066400	0.84043600
C	-0.11024800	-0.65648500	0.60937300
C	-0.14149600	0.88477200	0.52335400
C	0.82193900	1.40196500	-0.57742000
C	2.25040000	0.89066300	-0.34359200
C	2.28913900	-0.63972800	-0.23742100
H	-0.76535100	-1.01169000	1.40770100
H	0.45646600	1.04559000	-1.54571700

H	0.80762400	2.49631200	-0.60662900
H	2.89733000	1.23141700	-1.15764900
H	2.64691600	1.33375100	0.57934500
H	2.01024700	-1.08026400	-1.20007900
H	3.30613800	-0.97732200	-0.01687200
C	-1.50119100	1.50312600	0.30902500
H	-2.42093100	-0.89452400	-0.44082500
O	-2.52442700	0.92944900	0.01058800
H	1.30856300	-2.24279100	0.86183000
H	1.65385500	-0.80922900	1.82949200
H	0.21546200	1.29005300	1.48339000
O	-0.57596800	-1.24320800	-0.61787000
O	-1.92343600	-1.73514200	-0.43815000
H	-1.51585200	2.60800400	0.42693400

R2-R

C	-2.31185600	-0.74620200	-0.98249100
C	-3.08724700	0.51509700	-1.37593900
C	-1.05305900	-0.43193200	-0.15927600
O	-1.07553000	-1.20750600	1.10760600
O	-2.04304200	-0.84181400	1.91773600
O	-3.36047800	1.36348700	-0.26811200
O	-4.24614000	0.64806800	0.62992500
H	-4.01990800	0.24594200	-1.88280700
H	-2.50147200	1.16003000	-2.03979300
H	-1.07289000	0.61027700	0.16271800
H	-3.59971000	0.27067500	1.25587500
H	-2.97160300	-1.38320400	-0.39168400
H	-2.05363500	-1.30796200	-1.88522800
C	0.26156200	-0.80227200	-0.83113600
H	0.26173100	-1.88671200	-0.99444200
H	0.25971500	-0.34192500	-1.82698300
C	1.53849200	-0.39229300	-0.07691300
C	2.77496800	-1.06006400	-0.70697200
C	1.73233300	1.13497700	-0.02004200
H	1.45181700	-0.76114100	0.95344000
C	4.07385600	-0.67480300	0.01447600
H	2.84371600	-0.76015900	-1.76210100
H	2.65047200	-2.14852800	-0.70065300
C	3.02851200	1.52585900	0.70541900
H	1.76134400	1.52659700	-1.04660600
H	0.88086500	1.61545500	0.47225900
C	4.25397900	0.84779900	0.07901000
H	4.93073600	-1.13872200	-0.48438800

H	4.05093600	-1.07913200	1.03447600
H	3.14793300	2.61383400	0.69377600
H	2.95216900	1.23272700	1.76021800
H	5.15697600	1.09935700	0.64430800
H	4.40308600	1.23771000	-0.93643800
TS			
C	4.17129500	-0.70590700	0.83044900
C	2.82651000	-1.32914500	0.43120800
C	1.74956300	-0.26541100	0.14817800
C	2.25684800	0.73309100	-0.90931700
C	3.60057500	1.36294400	-0.51291300
C	4.66385800	0.29551500	-0.22276200
H	2.96612400	-1.94509000	-0.46848000
H	2.47795500	-2.00563700	1.21932800
H	4.05630300	-0.19022600	1.79238100
H	4.91730100	-1.49193600	0.98648900
H	2.37298000	0.20656300	-1.86744800
H	1.51885500	1.52415400	-1.07474100
H	3.94454400	2.03703400	-1.30410000
H	3.45618100	1.98184400	0.38171500
H	4.89881200	-0.24224800	-1.15074100
H	5.59492200	0.76721300	0.10770900
H	1.57135500	0.28829200	1.07846000
C	0.42324200	-0.93525900	-0.25133600
H	0.20323500	-1.74982800	0.44860100
H	0.52479400	-1.39404400	-1.24257200
C	-0.78832300	-0.00873500	-0.27649000
H	-0.59882900	0.88767500	-0.87615300
C	-2.07047600	-0.71246900	-0.78528500
H	-1.95761800	-0.93686200	-1.85209600
H	-2.18208900	-1.66025800	-0.25095200
C	-3.30760500	0.12254300	-0.53946300
H	-3.63378000	0.80759300	-1.32547300
H	-2.84080500	0.95399300	0.41889000
O	-1.02567600	0.41044700	1.07212800
O	-1.95469200	1.47656700	1.03319100
O	-4.33684900	-0.68108200	-0.07624400
O	-5.53503700	0.12522000	0.03439700
H	-5.62513000	0.16954500	0.99779300
P			
C	3.64126900	-0.44858700	0.83242400
C	2.33436400	-1.22145500	0.60691600
C	1.17367800	-0.30063700	0.18674200
C	1.57393900	0.52880500	-1.04781700

C	2.87939000	1.30695600	-0.82462700
C	4.02623400	0.38041800	-0.39987500
H	2.49266700	-1.97920800	-0.17384500
H	2.05992200	-1.76670100	1.51678100
H	3.51828700	0.22088200	1.69326100
H	4.44756500	-1.14278400	1.09107000
H	1.69917900	-0.14736900	-1.90588000
H	0.77570900	1.22883900	-1.31325700
H	3.14876600	1.85271600	-1.73484000
H	2.71718000	2.06285600	-0.04590600
H	4.26978800	-0.29672800	-1.22924600
H	4.93071400	0.96345000	-0.19814500
H	0.97688100	0.39272700	1.01362400
C	-0.10995000	-1.11593800	-0.03655000
H	-0.23606200	-1.82335800	0.79235100
H	-0.01232200	-1.71870900	-0.94864800
C	-1.39046800	-0.27956000	-0.12749400
H	-1.27214600	0.53409000	-0.84609300
C	-2.59722100	-1.14842400	-0.51235000
H	-2.42860700	-1.63757600	-1.48139900
H	-2.70625200	-1.97356100	0.20680000
C	-3.94557200	-0.47978900	-0.58586800
H	-4.75265400	-1.13062200	-0.98348900
O	-4.21015200	0.65317300	-0.25464400
O	-1.59808900	0.31800900	1.16202800
O	-1.97207000	1.70637300	1.00299800
H	-2.90011600	1.60988000	0.71479300

R3-R

C	1.25881000	0.67051300	-0.29331200
C	0.18923200	-0.05456400	0.54206300
C	2.64849300	0.62488700	0.36131100
O	3.65724800	0.12984400	-0.61017300
O	3.46431200	-1.12227800	-0.95697300
O	0.64230900	-1.37424600	0.88508500
O	0.78845100	-2.15196800	-0.33319000
H	0.12321300	0.40376200	1.53880300
H	2.66042400	-0.11386400	1.16436200
H	1.73970700	-2.02779800	-0.51204500
H	1.30915100	0.18933200	-1.27214300
H	0.96387200	1.70761000	-0.46175600
C	3.18582600	1.96463700	0.82700600
H	4.17675000	1.85861100	1.27225900
H	3.24800000	2.66943200	-0.00587700

H	2.51348000	2.38301600	1.58001700
C	-1.21329700	-0.06834300	-0.09330000
C	-2.18607800	-0.91985100	0.74527300
C	-1.77958600	1.35247400	-0.28959500
H	-1.11407400	-0.54072400	-1.07822200
C	-3.59726500	-0.94366700	0.14341500
H	-2.23476100	-0.50576500	1.76240900
H	-1.79398500	-1.93347300	0.84055300
C	-3.19710200	1.33136400	-0.88298700
H	-1.80335500	1.86665200	0.68179700
H	-1.13029300	1.94462600	-0.94114200
C	-4.15533100	0.47177000	-0.04961400
H	-4.26323200	-1.53138900	0.78328200
H	-3.56728900	-1.45653500	-0.82649000
H	-3.57710600	2.35463500	-0.96806400
H	-3.14986100	0.93121600	-1.90385400
H	-5.14032900	0.43403900	-0.52595600
H	-4.30108600	0.94048500	0.93250400
TS			
C	-3.25795400	-0.56816100	-1.18429900
C	-1.87723800	-1.01309700	-0.68274600
C	-1.03541100	0.18694200	-0.20465300
C	-1.80102200	0.99750300	0.87069600
C	-3.18360000	1.43386700	0.36574100
C	-4.01413200	0.23870200	-0.12020300
H	-1.99674500	-1.71908300	0.14542300
H	-1.33949600	-1.54343200	-1.47530300
H	-3.13640900	0.04629500	-2.08593600
H	-3.84222200	-1.44392200	-1.48332100
H	-1.91181700	0.37490800	1.76490400
H	-1.21643400	1.87451000	1.16657900
H	-3.71392000	1.96826300	1.16055000
H	-3.05937600	2.14737300	-0.45930700
H	-4.24211800	-0.41301500	0.73283700
H	-4.97498400	0.58228400	-0.51623800
H	-0.86874100	0.84554300	-1.06736000
C	0.35460500	-0.21940100	0.26702300
H	0.96118100	-0.90278100	-0.70075500
C	1.40248600	0.87296600	0.46743600
H	1.06467200	1.76731600	-0.06315900
H	1.47680300	1.11000600	1.53378700
C	2.81219800	0.50863600	-0.06039500
H	3.27669800	-0.21690400	0.61174000
O	2.68083200	-0.09441700	-1.35691300

O	1.99362800	-1.32359500	-1.21255400
O	0.21465400	-1.14732400	1.30100000
O	1.51219900	-1.60987200	1.75069400
H	1.83436700	-2.08139000	0.96076200
C	3.71124700	1.71879200	-0.25082000
H	3.84361600	2.24186300	0.69924500
H	4.69226100	1.40980800	-0.61598200
H	3.27508900	2.41353600	-0.97295800

P

C	3.49977200	-0.72174700	0.87845800
C	2.03069700	-1.11143900	0.66537600
C	1.15588000	0.11705000	0.36503100
C	1.72049000	0.90831500	-0.84470000
C	3.18908800	1.29568100	-0.62321400
C	4.05624300	0.06863000	-0.31372600
H	1.94331300	-1.81212100	-0.17039600
H	1.64488100	-1.63257500	1.54730600
H	3.58896700	-0.11414800	1.78856500
H	4.09827800	-1.62197500	1.04858600
H	1.63608400	0.27877500	-1.73810800
H	1.11961100	1.80497800	-1.02974200
H	3.56752300	1.81617700	-1.50862200
H	3.25425200	2.00778600	0.20937200
H	4.08614600	-0.58266900	-1.19631900
H	5.08799900	0.37615800	-0.11708100
H	1.16277700	0.78933000	1.23324300
C	-0.29019400	-0.26028900	0.06858800
C	-1.34990300	0.73283500	0.51583100
H	-1.42298900	0.62736300	1.60750200
H	-0.96519000	1.74551100	0.34714100
C	-2.75791900	0.59479300	-0.08600500
O	-0.55246200	-1.30061800	-0.50760000
O	-3.35891200	-0.64806500	0.30536800
O	-3.26808700	-1.59845400	-0.78094100
H	-2.31434200	-1.81138500	-0.74904200
H	-2.70740200	0.60258000	-1.17785200
C	-3.68462700	1.70434500	0.40483500
H	-3.28320500	2.69125600	0.15841000
H	-4.66583000	1.59791000	-0.05964600
H	-3.81235800	1.64510700	1.48922600

R4

C	2.28433000	-1.38460300	-0.62618900
C	0.75156300	-1.35651000	-0.69813000

C	0.12746500	-0.67537600	0.53648100
C	0.74414300	0.71409200	0.79564600
C	2.27168800	0.70849600	0.79274000
C	2.86017500	0.02530300	-0.44893100
H	0.43365900	-0.81903100	-1.59514800
H	0.36222400	-2.37424900	-0.78843700
H	2.60358000	-2.01702300	0.21295000
H	2.68763500	-1.84482700	-1.53309300
H	0.34875800	1.14240200	1.71822400
H	2.63062200	1.73718000	0.88666600
H	2.59436400	0.17594500	1.69546400
H	2.63274400	0.62687600	-1.33549500
H	3.95026800	-0.01086800	-0.36430400
H	0.39638000	-1.26539700	1.42421800
C	-1.41518100	-0.61827700	0.49724800
H	-1.75670900	0.14222200	1.20455700
O	-1.78416500	-0.16457200	-0.81980400
O	-2.92600900	0.71234700	-0.70877900
H	-2.47300600	1.57366300	-0.70099500
O	0.32095500	1.65762200	-0.26129900
O	-0.69147400	2.40969200	0.11358800
C	-2.10695300	-1.94240900	0.80700400
H	-1.88104500	-2.27585200	1.82447000
H	-1.80006800	-2.72285300	0.10709800
H	-3.18724700	-1.81319800	0.71896700
TS			
C	-2.12991600	-1.07822800	-0.77817700
C	-0.65343500	-0.95706400	-0.40860200
C	-0.13374100	0.51408600	-0.45669000
C	-1.05095700	1.44881800	0.35123000
C	-2.51577500	1.32681300	-0.09245500
C	-3.02221100	-0.11634100	0.01788100
H	-0.03181600	-1.57835400	-1.06010900
H	-0.97472600	1.19698900	1.41415800
H	-0.71370100	2.48315500	0.24230000
H	-3.13826900	1.99332500	0.51212000
H	-2.61034200	1.66935700	-1.13139400
H	-3.03298500	-0.42004900	1.06946200
H	-4.05274000	-0.18664000	-0.34301100
C	1.31849800	0.53531700	0.00026600
H	1.25452000	-0.46886900	0.88889400
C	1.93697400	1.75148100	0.64759100
H	1.40274300	2.03216600	1.55576300
H	2.97534000	1.54094600	0.90229000

H	1.91910000	2.60183000	-0.04622900
O	2.07155200	-0.01282100	-1.04705300
O	3.35397000	-0.46855200	-0.53455600
H	3.12722800	-1.38478500	-0.30888000
H	-2.44209900	-2.11563600	-0.63103400
H	-2.21872900	-0.86705000	-1.85026000
H	-0.15615300	0.81528700	-1.51490600
O	-0.51481800	-1.43872800	0.93423400
O	0.86379600	-1.56252000	1.22419800

P

C	-1.92446400	0.34702800	1.02392000
C	-0.47366100	0.71950800	0.68812400
C	0.33246900	-0.56576500	0.36329100
C	-0.32329700	-1.30771800	-0.83244600
C	-1.78616300	-1.65893100	-0.52398700
C	-2.59737900	-0.41921400	-0.12429300
H	-0.01794500	1.26245500	1.52014600
H	-0.27161800	-0.65161100	-1.70619000
H	0.23794400	-2.21571200	-1.07372100
H	-2.23736500	-2.14075300	-1.39700100
H	-1.81539900	-2.39536500	0.28981500
H	-2.69167500	0.24854200	-0.98614700
H	-3.61192700	-0.70926900	0.16525100
C	1.81846600	-0.38088900	0.08135100
H	1.26836600	2.11931400	-0.57218100
C	2.72515900	-1.52602900	0.48321800
H	2.29101600	-2.49588900	0.22854600
H	3.70067800	-1.41106900	0.01283900
H	2.85057000	-1.50722500	1.57180900
O	2.28585000	0.60843700	-0.45179300
H	-2.47085100	1.26657300	1.24615500
H	-1.92706200	-0.26311200	1.93558800
H	0.25540200	-1.22241300	1.24035600
O	-0.54040900	1.61784300	-0.42666700
O	0.48119600	2.63216900	-0.30033200

R5-R

C	1.81202000	-2.03559700	-0.67492800
C	0.43714200	-1.37880100	-0.86922700
C	-0.01579400	-0.62257500	0.39556300
C	1.05099600	0.39000200	0.83631400
C	2.43011100	-0.24405500	1.01304700
C	2.87339500	-1.01892700	-0.23571200
H	0.46708600	-0.68066500	-1.71042900

H	-0.30224700	-2.14139000	-1.12597900
H	1.73113200	-2.82648100	0.08243700
H	2.12293000	-2.52559000	-1.60252000
H	0.74280200	0.92502000	1.73789900
H	3.14445500	0.54250200	1.26568300
H	2.37401400	-0.91625300	1.87716000
H	3.05821300	-0.31054200	-1.05022100
H	3.82513100	-1.51991100	-0.03631400
H	-0.03853100	-1.34350700	1.22524400
C	-1.45710100	-0.05991800	0.31078100
H	-1.63085500	0.62076500	1.15098500
O	-1.69231500	0.68962100	-0.89347000
O	-1.52779000	2.10277600	-0.63317300
H	-0.59275800	2.22323500	-0.86198500
O	1.14405100	1.43762500	-0.21388300
O	1.92908300	2.43478900	0.14319400
C	-2.52877000	-1.15922100	0.32911800
H	-2.29482300	-1.85939800	1.13872200
H	-2.47003000	-1.72734100	-0.60440900
C	-3.94367200	-0.60344600	0.50336900
H	-4.17043000	0.11646300	-0.28462400
H	-4.68614300	-1.40436900	0.46059400
H	-4.05414400	-0.09579100	1.46647500
TS			
C	2.31563200	-1.67240600	-0.45800600
C	0.88006300	-1.25782900	-0.80567800
C	2.58525900	0.38938800	0.97389800
C	3.19371700	-0.45370800	-0.15341100
H	0.88697100	-0.66120700	-1.72214100
H	0.26863900	-2.13892200	-1.01170600
H	2.30539000	-2.34284800	0.41195100
H	2.73982200	-2.24744500	-1.28674700
H	3.16327700	1.30259100	1.13942500
H	2.60781600	-0.17717600	1.91244200
H	3.29036100	0.16118600	-1.05403700
H	4.20393300	-0.76763700	0.12649900
C	-1.17476800	0.08041400	0.00160800
H	-0.85625800	1.32118500	-0.37446000
O	1.12237000	1.70864300	-0.37990600
O	-0.15697100	2.31345100	-0.41489200
C	0.23114800	-0.43518900	0.32536900
H	0.16785800	-1.06422200	1.22519300
C	1.13266400	0.78229600	0.71023600
H	0.71520700	1.29376800	1.58414500

C	-2.14895800	0.26719300	1.15583900
H	-2.95627400	0.92717200	0.83255800
H	-1.62049300	0.78815700	1.95931000
C	-2.74459900	-1.05391100	1.66870300
H	-1.96952700	-1.73720800	2.02695900
H	-3.42975800	-0.86307200	2.49847100
H	-3.30111000	-1.55949000	0.87771800
O	-1.68586200	-0.60828700	-1.10130500
O	-2.93162700	0.00855100	-1.52434700
H	-2.62008800	0.50882300	-2.29332600

P

C	2.19089000	-1.59576200	-0.45550200
C	0.73345200	-1.31141000	-0.83845100
C	2.16862400	0.30350000	1.20439500
C	2.90294700	-0.32133300	0.01221000
H	0.70715800	-0.66781800	-1.71780500
H	0.22712300	-2.24420700	-1.11028600
H	2.22350200	-2.34878500	0.34364600
H	2.71197700	-2.03196000	-1.31331000
H	2.63081800	1.24812900	1.50322700
H	2.24517800	-0.37437600	2.06492900
H	2.94182700	0.39889000	-0.80819200
H	3.93495600	-0.54780300	0.29849600
C	-1.45380400	-0.23083500	-0.15164300
C	-0.05393600	-0.63809400	0.31104300
H	-0.16144900	-1.37374900	1.11762100
C	0.67678500	0.57835400	0.96311500
H	0.20447600	0.77246900	1.93404700
C	-2.62673900	-0.67472100	0.69977600
H	-2.42432600	-0.34986700	1.72927100
H	-2.59764000	-1.77212700	0.74205300
C	-3.98210000	-0.17296500	0.21252700
H	-4.18110600	-0.51516300	-0.80455900
H	-4.78300200	-0.53315100	0.86215000
H	-4.01169800	0.91811600	0.19989500
O	-1.61822900	0.42078800	-1.16586300
O	0.41767200	1.82907800	0.32327500
O	0.82705700	1.78698800	-1.07116400
H	-0.01650700	1.50617300	-1.47598800

R6-R

C	1.93739100	-1.55134300	-0.78692500
C	0.44319400	-1.20385500	-0.84713700
C	-0.03930700	-0.59724700	0.48504700

C	0.79715800	0.65158100	0.85696600
C	2.29116700	0.30247200	0.90406700
C	2.78444100	-0.33057300	-0.40482500
H	0.26977900	-0.48392600	-1.65140700
H	-0.14489600	-2.09378300	-1.09064500
H	2.09647800	-2.35160200	-0.05184500
H	2.26164400	-1.94841400	-1.75367000
H	0.47656500	1.04279600	1.82859100
H	2.85018000	1.21333200	1.13103600
H	2.45054700	-0.39310700	1.73672700
H	2.73143800	0.41528100	-1.20423000
H	3.83679500	-0.61361700	-0.30509900
H	0.18646600	-1.32775000	1.27367400
C	-1.57680900	-0.39857400	0.55984100
H	-1.82619400	0.55229500	1.03056400
O	0.68943300	1.71929700	-0.09218300
O	-0.50283600	2.49168900	0.20033600
H	-1.11353300	2.12833700	-0.46887000
O	-2.19330600	-0.34179300	-0.79136200
O	-2.07127900	0.81283800	-1.40386300
C	-2.30421800	-1.55186800	1.23237100
H	-2.00829900	-1.60480400	2.28245000
H	-2.05554100	-2.50408100	0.75705000
H	-3.38562200	-1.41076000	1.18581800
TS			
C	1.88821700	-1.91908500	0.08280200
C	0.52094100	-1.73908400	-0.60624300
C	-0.31282600	-0.70513900	0.18513400
C	0.47235900	0.58982100	0.28260500
C	1.78815900	0.47221100	1.01749100
C	2.64458400	-0.59556000	0.28777300
H	0.65593000	-1.39310400	-1.63582700
H	-0.00186700	-2.69862400	-0.64784600
H	1.72905200	-2.39939600	1.05673900
H	2.50632900	-2.60665500	-0.50294500
H	-0.52101600	1.35596600	0.81635400
H	2.30231200	1.43420400	1.03618800
H	1.59767900	0.17046300	2.05213000
H	2.94018800	-0.18987300	-0.68445100
H	3.56418600	-0.77312200	0.85374900
H	-0.41264200	-1.07922200	1.21267200
C	-1.75115100	-0.40977200	-0.29273600
H	-1.73144400	0.12491100	-1.24759200
C	-2.66225900	-1.62360000	-0.36035100

H	-2.33279800	-2.30570900	-1.14650700
H	-2.66826000	-2.16188400	0.59093800
O	-2.33883800	0.45344100	0.70312000
O	-1.66325800	1.69765200	0.71827900
H	-3.68311000	-1.30962500	-0.58503200
O	0.58215800	1.13136200	-1.00576600
O	0.97389700	2.52669300	-0.89927500
H	0.09565600	2.93974000	-0.87828300

P

C	-2.48046000	1.16383300	0.05295000
C	-1.03476900	1.39727700	-0.40460800
C	-0.03118200	0.42315900	0.26851400
C	-0.52068000	-1.01250300	0.06053700
C	-1.95359500	-1.27606800	0.48516800
C	-2.92371700	-0.28266600	-0.18456600
H	-0.96987200	1.27742300	-1.49356600
H	-0.74083100	2.42560400	-0.18486400
H	-2.56527000	1.40140300	1.12073700
H	-3.14795200	1.85295600	-0.47318300
H	-2.19530200	-2.31229600	0.24465000
H	-2.01011400	-1.15633900	1.57470400
H	-2.95471000	-0.48510300	-1.26149600
H	-3.93667000	-0.44616200	0.19360200
H	-0.07653500	0.60112500	1.35278700
C	1.43068100	0.62738300	-0.17001300
C	1.95802800	2.04330700	0.04955500
H	1.43953100	2.77562000	-0.57350900
H	1.85278600	2.34022300	1.09712000
O	0.15376600	-1.89208500	-0.44024700
H	1.54762500	0.34601500	-1.21904600
O	2.25865100	-0.26205900	0.60655200
O	2.83965900	-1.26590500	-0.25591900
H	2.05598500	-1.82925700	-0.40242400
H	3.01889500	2.06684900	-0.20380800

R7-R

C	-2.10605800	-1.70889700	0.79513700
C	-0.70009900	-1.12175700	0.98390400
C	-0.14603100	-0.57118300	-0.34514500
C	-1.10488400	0.48849500	-0.94160100
C	-2.51286600	-0.09698300	-1.11683300
C	-3.06950400	-0.67977800	0.18990000
H	-0.74034600	-0.31256100	1.71789700
H	-0.02289100	-1.87948800	1.38887100

H	-2.04946400	-2.58759100	0.13877700
H	-2.48821000	-2.06386000	1.75713200
H	-0.72556600	0.83842100	-1.90771100
H	-3.16671000	0.68878500	-1.50256500
H	-2.46073100	-0.88148400	-1.88129700
H	-3.22977900	0.13420000	0.90398800
H	-4.04693600	-1.13576900	0.00485000
H	-0.15797500	-1.39841200	-1.06743800
C	1.33937100	-0.12655300	-0.27036600
H	1.49593500	0.81337300	-0.80089600
O	-1.27656500	1.63996600	-0.10538700
O	-0.19109900	2.57018700	-0.35181500
H	0.38464000	2.37516700	0.41192700
O	1.75151700	0.14017700	1.13117900
O	1.42523500	1.32860700	1.58427500
C	2.32040800	-1.19180900	-0.75334000
H	2.02756600	-1.45146200	-1.77578600
H	2.18293500	-2.09772700	-0.15229600
C	3.78621500	-0.75072700	-0.72893300
H	4.11081800	-0.50860900	0.28458800
H	4.43363500	-1.54406100	-1.10969300
H	3.94107300	0.13474000	-1.35269900
TS			
C	1.87527500	-2.18122100	0.06013900
C	0.51075200	-1.78071300	-0.53527400
C	-0.07022300	-0.59910800	0.27600600
C	0.93435300	0.53930300	0.26859400
C	2.26241900	0.21073800	0.91158800
C	2.86329200	-1.00702600	0.16218200
H	0.62376500	-1.48766500	-1.58372700
H	-0.16966100	-2.63592400	-0.50476200
H	1.71060900	-2.60318600	1.05986500
H	2.31763200	-2.98031400	-0.54294300
H	0.13632000	1.48449900	0.84372100
H	2.93478700	1.06822700	0.85859900
H	2.10230200	-0.02862900	1.96746600
H	3.14945700	-0.68228100	-0.84271800
H	3.77877900	-1.32980300	0.66739900
H	-0.15261600	-0.92559200	1.32094900
C	-1.46725000	-0.06077800	-0.10710300
H	-1.42562000	0.44617700	-1.07725100
C	-2.58781700	-1.09444400	-0.08071400
H	-2.32444600	-1.89612900	-0.77710000
H	-2.62773900	-1.54292600	0.91830000

O	-1.80845200	0.91219500	0.90312900
O	-0.93186300	2.02011300	0.82023300
O	1.03747300	1.02120200	-1.04392300
O	1.67493500	2.32687500	-1.01872800
H	0.88706800	2.89002600	-0.95217200
C	-3.95542100	-0.51350400	-0.45358000
H	-4.72640700	-1.28741100	-0.42373500
H	-4.24187300	0.28232500	0.23533500
H	-3.94137600	-0.09396000	-1.46394000

P

C	2.50051700	-1.57289800	0.05596700
C	1.02785400	-1.48801400	-0.36644300
C	0.28308800	-0.30098800	0.30274900
C	1.07585200	0.98220000	0.03674200
C	2.54103600	0.93108800	0.42937900
C	3.24990100	-0.26857900	-0.22991900
H	0.96272400	-1.37892700	-1.45657400
H	0.52159600	-2.42169900	-0.11471100
H	2.55632400	-1.79886900	1.12813400
H	2.98363500	-2.40611000	-0.46334100
H	3.00049000	1.88157000	0.15472700
H	2.59552200	0.82748500	1.52070000
H	3.29923200	-0.10289400	-1.31246500
H	4.28248400	-0.32692200	0.12527800
H	0.32435600	-0.45832200	1.39031000
C	-1.20188500	-0.17934200	-0.09037100
C	-2.03537000	-1.43191700	0.20175200
H	-1.60254000	-2.28806000	-0.32440700
H	-1.96740400	-1.65225900	1.27393700
O	0.60572400	1.97407000	-0.48735000
C	-3.50220300	-1.26964700	-0.20436600
H	-4.07790600	-2.16181400	0.05524100
H	-3.94734100	-0.41153700	0.30060200
H	-3.59757800	-1.11063400	-1.28268400
H	-1.28873800	0.08391900	-1.14803500
O	-1.77091200	0.90645400	0.66989800
O	-2.13809800	1.98991900	-0.21414500
H	-1.25130500	2.35010800	-0.40687000

R8-R

C	-2.23438400	-1.85647400	0.91641700
C	-0.97104400	-1.00940300	1.12538300
C	-0.39066000	-0.53701800	-0.22236500
C	-1.45069800	0.25282900	-1.02880400

C	-2.71698800	-0.59201600	-1.22540500
C	-3.29282800	-1.09875900	0.10449800
H	-1.21638000	-0.13625400	1.73576200
H	-0.21920100	-1.57873600	1.67977300
H	-1.96856000	-2.78537600	0.39430800
H	-2.64339600	-2.15202600	1.88741600
H	-1.04339400	0.54352500	-2.00311000
H	-3.45239200	0.01040500	-1.76394700
H	-2.46011100	-1.44296200	-1.86764900
H	-3.65577500	-0.24456000	0.68484300
H	-4.15752000	-1.74135600	-0.08769100
H	-0.19451500	-1.43577300	-0.82210500
C	0.98593200	0.17092900	-0.10016700
H	1.03343200	1.05082400	-0.74271400
O	-1.89335600	1.44893600	-0.37454500
O	-0.96086700	2.52108300	-0.66635900
H	-0.43659200	2.52709400	0.15699800
O	1.20917700	0.67857100	1.27784700
O	0.64436700	1.83529200	1.53672300
C	2.17281900	-0.75345300	-0.35550400
H	2.02553000	-1.19721700	-1.34655100
H	2.13939400	-1.58222700	0.36220000
C	3.54039800	-0.06227500	-0.30242500
H	3.67910400	0.39182300	0.68268900
H	3.55347800	0.76091300	-1.02674600
C	4.69650600	-1.02216800	-0.59526800
H	5.65829800	-0.50499800	-0.55490200
H	4.72815300	-1.83774300	0.13360600
H	4.60156700	-1.46956900	-1.58967400
TS			
C	1.90721800	2.37998200	-0.07703300
C	0.60403200	1.78281000	0.49068100
C	0.23704600	0.51138900	-0.30964800
C	1.40512200	-0.45661200	-0.25045100
C	2.68455100	0.06256400	-0.86562100
C	3.06769600	1.37188900	-0.12768100
H	0.73008500	1.52927100	1.54792000
H	-0.19931200	2.52129500	0.42352500
H	1.70869900	2.75406400	-1.08959500
H	2.20265300	3.24831800	0.52017500
H	0.78091600	-1.52460100	-0.82536100
H	3.47963000	-0.67909400	-0.77625600
H	2.52025200	0.25553400	-1.93032200
H	3.37114900	1.11314900	0.89120300

H	3.93653900	1.82407800	-0.61572600
H	0.13591100	0.80279800	-1.36323700
C	-1.07069800	-0.23086200	0.04839400
H	-0.97905200	-0.70745100	1.03028800
C	-2.33606400	0.61490200	-0.03188800
H	-2.22056900	1.46716600	0.64606300
H	-2.42205200	1.02614200	-1.04499300
O	-1.22639700	-1.26377200	-0.94846400
O	-0.19107200	-2.21969600	-0.81773800
O	1.54299400	-0.89323600	1.07466500
O	2.37379700	-2.08538600	1.09477100
H	1.68400800	-2.76418200	1.01889000
C	-3.61354900	-0.15415000	0.32909400
H	-3.70935700	-1.01736800	-0.33430900
H	-3.51410900	-0.55527500	1.34468000
C	-4.87042400	0.71506200	0.24086200
H	-5.01284800	1.10298000	-0.77257100
H	-5.76489000	0.14501300	0.50503300
H	-4.81179700	1.57303200	0.91846200
P			
C	2.47260300	-1.96476100	0.03892400
C	1.06443800	-1.54397600	-0.40172500
C	0.59126900	-0.23467000	0.28727300
C	1.65396200	0.84293000	0.05394200
C	3.06272500	0.46040800	0.46888900
C	3.49968300	-0.85556100	-0.20540000
H	1.04646000	-1.39943300	-1.48948500
H	0.35812900	-2.34623300	-0.18083700
H	2.45595000	-2.21907900	1.10601700
H	2.76709200	-2.87413600	-0.49360100
H	3.72789500	1.28884500	0.22148700
H	3.07115300	0.32806500	1.55831000
H	3.60679600	-0.68520700	-1.28297400
H	4.48583300	-1.14941400	0.16468000
H	0.58278800	-0.41845500	1.37128900
C	-0.82540400	0.22068100	-0.11412500
C	-1.92010400	-0.81496000	0.16214000
H	-1.68327400	-1.74836700	-0.35927600
H	-1.92079800	-1.04445000	1.23553400
O	1.42762100	1.92003200	-0.46389100
C	-3.31458400	-0.34005300	-0.26214100
H	-3.52387100	0.61428100	0.22661200
H	-3.31422200	-0.14080700	-1.34030900
H	-0.84218700	0.50593400	-1.16935000

O	-1.14195500	1.40049700	0.65385200
O	-1.24365500	2.54694900	-0.22123500
H	-0.29601500	2.69802200	-0.40181000
C	-4.41209700	-1.35360700	0.07251800
H	-5.39448200	-0.98988900	-0.24014800
H	-4.23988100	-2.31278700	-0.42694800
H	-4.45839600	-1.54529400	1.14922800

R9-R

C	-1.00016600	-0.25336100	1.25086700
C	-2.11803700	-1.13124100	0.68529800
O	-0.15900900	0.71528200	-0.85579000
O	-0.74612500	1.86150200	-0.59552100
O	-2.62004400	-0.70976500	-0.57755200
O	-3.30247000	0.55189000	-0.38431900
H	-2.94003600	-1.18143700	1.40787700
H	-1.77771400	-2.15113500	0.48506700
H	-2.64105700	1.17938100	-0.72711000
H	-1.40605800	0.73203700	1.48823100
H	-0.67410300	-0.69446100	2.19893700
C	0.24539400	-0.05085300	0.37593500
C	1.30098300	0.78515100	1.11503900
C	0.82859100	-1.34151900	-0.20907800
C	2.59084900	0.99130700	0.31025700
H	1.53149000	0.26407500	2.05143900
H	0.85060800	1.74394600	1.38429400
C	2.13200000	-1.12094900	-0.99320600
H	1.01122900	-2.02505800	0.62881300
H	0.08121500	-1.81234800	-0.85175500
C	3.16948800	-0.34341800	-0.17513500
H	3.31911700	1.52474600	0.92836900
H	2.38243900	1.63349800	-0.55196700
H	2.53314500	-2.09120900	-1.30104800
H	1.90722800	-0.56952600	-1.91188900
H	4.06871600	-0.17096100	-0.77408900
H	3.48035900	-0.94352200	0.69042300

TS

C	1.02662300	-0.78034800	-1.00343500
C	2.36917400	-1.30794000	-0.48069400
C	3.38105400	-0.17274000	-0.28082300
C	2.81564400	0.91354900	0.64296900
C	1.46235500	1.43888500	0.14220900
C	0.44057600	0.31793400	-0.09965500
H	4.31565700	-0.56626700	0.13087300

H	2.20043500	-1.82191200	0.47077700
H	2.76378600	-2.05330800	-1.17825400
H	1.16101000	-0.35040100	-2.00351900
H	0.31220300	-1.59994600	-1.09538000
H	2.68537300	0.50302800	1.64887300
H	3.51877100	1.74796200	0.72876500
H	1.03857400	2.14864400	0.85910400
H	1.59599400	1.97841400	-0.80282800
H	3.63041000	0.26884400	-1.25514600
C	-0.89179300	0.89923900	-0.65358300
C	-2.05861200	-0.05163700	-0.50520800
H	-0.75205900	1.18221700	-1.70304400
H	-1.12661400	1.80685200	-0.09103900
H	-2.28015000	-0.73990600	-1.32349800
H	-1.57951900	-0.85623100	0.46837300
O	0.14862900	-0.17990100	1.22827300
O	-0.69826400	-1.30986000	1.13896500
O	-3.18097600	0.64714800	-0.08797300
O	-4.30766500	-0.26312700	-0.06820900
H	-4.44778800	-0.34372500	0.88681300
P			
C	-0.60144900	-1.38476100	-0.12344900
C	-1.92969100	-1.16571500	-0.86301200
C	-2.88416800	-0.28082300	-0.05129600
C	-2.22062500	1.05252600	0.31643500
C	-0.89903600	0.84216600	1.06705000
C	0.07490900	-0.06386300	0.29332100
H	-3.80363000	-0.10224800	-0.61733900
H	-1.72672900	-0.69417800	-1.82882900
H	-2.39070300	-2.13627400	-1.07192500
H	-0.78034900	-1.96744300	0.78778900
H	0.08893800	-1.96152800	-0.74681600
H	-2.02079200	1.62334400	-0.59557300
H	-2.89445300	1.65880500	0.92984300
H	-0.40554100	1.80000600	1.24695000
H	-1.09286000	0.38624700	2.04608100
H	-3.17926800	-0.80777000	0.86619400
C	1.35459400	-0.31416900	1.14764100
C	2.50431700	-0.89088300	0.35494900
H	1.10207700	-0.97226800	1.98241000
H	1.68332200	0.64768600	1.54943600
H	2.73813300	-1.96390000	0.50638200
O	3.16801300	-0.24008900	-0.42113300
O	0.42916500	0.52957400	-0.96835600

O	1.17837900	1.75175800	-0.74753400
H	2.08428700	1.39270900	-0.80825000

R10-R

C	-0.87635900	-0.44122400	0.91683000
C	-2.00700300	-0.92204000	-0.00322300
O	0.29477100	1.09952100	-0.62074400
O	-0.19096200	2.14970200	0.00238100
O	-2.30536900	0.01409800	-1.05032300
O	-2.84830200	1.22152400	-0.46157600
H	-1.67295800	-1.78289900	-0.59169200
H	-2.07873500	1.81634900	-0.51091200
H	-1.21705800	0.43928900	1.46669300
H	-0.69082900	-1.22177600	1.66149600
C	0.47448300	-0.10054700	0.26854200
C	1.51058700	0.26968800	1.34149900
C	1.00063000	-1.16111500	-0.70511800
C	2.90001400	0.58073300	0.77006800
H	1.57967000	-0.57791800	2.03292800
H	1.12391200	1.11808000	1.91175500
C	2.40282500	-0.84680400	-1.25052300
H	1.01724000	-2.11701400	-0.16793700
H	0.29492300	-1.26839100	-1.53181900
C	3.40475400	-0.55558100	-0.12757600
H	3.59650100	0.75843600	1.59492300
H	2.85598200	1.51001100	0.19232600
H	2.74473200	-1.68644200	-1.86312900
H	2.34116100	0.02078000	-1.91540000
H	4.38071700	-0.29945200	-0.55070700
H	3.55329700	-1.46033600	0.47684000
C	-3.26073300	-1.30851400	0.77816000
H	-4.03702300	-1.64519500	0.08847600
H	-3.04442600	-2.11761500	1.48124400
H	-3.64340400	-0.45223800	1.33506200

TS

C	-0.92412700	1.06314400	-0.50325200
C	-2.24632100	1.53971700	0.11199900
C	-3.41645300	0.63494600	-0.29366000
C	-3.13261100	-0.82809700	0.06955900
C	-1.80149900	-1.31354800	-0.52268900
C	-0.61804000	-0.40378300	-0.15914500
H	-4.34028000	0.96892000	0.18899500
H	-2.14835600	1.54637600	1.20226300
H	-2.43836000	2.57256000	-0.19535800

H	-0.97100700	1.14356300	-1.59583800
H	-0.09937000	1.69483300	-0.17518700
H	-3.09390000	-0.93109800	1.15831400
H	-3.94275200	-1.47472400	-0.28210700
H	-1.58034100	-2.32958000	-0.18146200
H	-1.86892300	-1.34962300	-1.61641300
H	-3.58200500	0.71747800	-1.37633400
C	0.69370300	-0.92273900	-0.82118600
C	1.96359600	-0.40672000	-0.16686900
H	0.69234200	-0.65271700	-1.88343600
H	0.69543900	-2.01318800	-0.75464900
H	1.44982600	-0.04501500	1.02209300
O	-0.46111500	-0.59683300	1.26848000
O	0.55170000	0.25641600	1.76100200
C	3.09542800	-1.37898500	0.07422900
H	3.45847600	-1.78172400	-0.88027900
H	3.92604800	-0.88647600	0.57780500
H	2.74873400	-2.20939000	0.69212000
O	2.32084500	0.78305600	-0.81392300
O	3.40145200	1.43090100	-0.08907900
H	2.90931600	2.13787300	0.35371500

P

C	-0.60458200	-1.29324600	-0.33979200
C	-1.99026700	-1.26150400	-1.00200900
C	-3.05845400	-0.72484800	-0.04117000
C	-2.66520200	0.65469400	0.50231500
C	-1.28758800	0.62853800	1.17684400
C	-0.19116500	0.06884600	0.25171800
H	-4.02684900	-0.67036300	-0.54821000
H	-1.94689200	-0.62618300	-1.89141500
H	-2.25108700	-2.26899500	-1.34165100
H	-0.60642100	-2.02450400	0.47735000
H	0.15112500	-1.61363700	-1.06338100
H	-2.64057500	1.37566100	-0.32053100
H	-3.41363700	1.01225900	1.21648800
H	-0.99354500	1.63560100	1.48155900
H	-1.32613300	0.01169700	2.08370500
H	-3.18243300	-1.42584800	0.79548300
C	1.15306800	-0.02165500	1.03308600
C	2.38510800	-0.21213100	0.15849300
H	1.07197900	-0.82152200	1.77262300
H	1.29059000	0.92090100	1.56901800
O	2.78764100	0.68689000	-0.55601600
O	-0.04696800	0.89287700	-0.91723200

O	0.45094000	2.20203800	-0.54327600
H	1.40756800	2.03907600	-0.66619000
C	3.10806300	-1.53720300	0.22629000
H	2.41629600	-2.35819200	0.01428600
H	3.93489600	-1.55024800	-0.48229500
H	3.48832300	-1.69908200	1.24071000

R11-R

C	-0.57374700	-0.20470300	0.94049300
C	-1.76705000	-0.56256900	0.04498400
O	0.76573000	1.13156700	-0.65132200
O	0.42199500	2.25198400	-0.05700500
O	-1.95719700	0.38066100	-1.02171000
O	-2.33793800	1.66020400	-0.45706000
H	-1.55287400	-1.47249400	-0.52708200
H	-1.49907700	2.15076800	-0.52353000
H	-0.79290100	0.72638300	1.46841700
H	-0.47889800	-0.98189900	1.70537300
C	0.80216300	-0.05556600	0.27269300
C	1.88717200	0.21198400	1.32741600
C	1.18163400	-1.20209200	-0.67128200
C	3.29963100	0.32793100	0.73974800
H	1.85509200	-0.61694100	2.04372900
H	1.61622300	1.11853000	1.87450300
C	2.60726400	-1.08384400	-1.23357400
H	1.08248800	-2.13574900	-0.10466400
H	0.46093100	-1.24522200	-1.49097800
C	3.64862000	-0.88883900	-0.12587400
H	4.02008600	0.43985100	1.55554500
H	3.36859900	1.23819200	0.13467300
H	2.83452000	-1.97784000	-1.82211900
H	2.64957000	-0.23567500	-1.92460700
H	4.64497400	-0.77055500	-0.56227700
H	3.68771100	-1.78714100	0.50456800
C	-3.05046600	-0.77961700	0.85535300
H	-2.85009900	-1.54811400	1.61081000
H	-3.27837600	0.14407300	1.39386900
C	-4.24491200	-1.18927100	-0.00867900
H	-5.14169100	-1.31583100	0.60308400
H	-4.45153500	-0.43022400	-0.76465500
H	-4.05731100	-2.13592400	-0.52555300

TS

C	-1.30127900	0.93815800	-0.70129300
C	-2.70804900	1.35708400	-0.25490600

C	-3.73644200	0.24948500	-0.51584800
C	-3.31216700	-1.06442800	0.15251600
C	-1.89673700	-1.48592200	-0.26840700
C	-0.85310500	-0.37997500	-0.04855100
H	-4.72314700	0.55365200	-0.15270100
H	-2.68557600	1.59281700	0.81382400
H	-2.99606300	2.27545800	-0.77624000
H	-1.28307700	0.79233900	-1.78794300
H	-0.57860900	1.72155000	-0.47552600
H	-3.33648400	-0.94323100	1.23981000
H	-4.01686600	-1.86499300	-0.09377000
H	-1.58437500	-2.37739800	0.28422600
H	-1.88461600	-1.74734500	-1.33310300
H	-3.83640000	0.09344300	-1.59848000
C	0.55189100	-0.85103900	-0.53329500
C	1.70555600	-0.05884500	0.05872900
H	0.58669700	-0.80060400	-1.62723700
H	0.67199900	-1.89770500	-0.24372000
H	1.07158500	0.46635100	1.11975200
O	-0.77427400	-0.26290900	1.39312500
O	0.09631400	0.79376000	1.74300700
C	2.92410500	-0.80507400	0.57194100
H	2.57771700	-1.54060200	1.30390400
H	3.56501600	-0.10026700	1.10435800
O	1.97141600	1.00996200	-0.80832000
O	2.91955300	1.92177300	-0.18874600
H	2.31981800	2.63018500	0.08797700
C	3.72902500	-1.49219300	-0.54484300
H	4.60389200	-1.99626300	-0.12643000
H	3.13153700	-2.24173900	-1.07109000
H	4.07608800	-0.75985300	-1.27640100
P			
C	-0.77438300	-1.30526300	-0.22085700
C	-2.11302300	-1.51438500	-0.94485900
C	-3.29580900	-1.05823700	-0.08136000
C	-3.12090900	0.39867500	0.36556100
C	-1.79084000	0.61242900	1.09967700
C	-0.58020700	0.14218400	0.27194500
H	-4.23225900	-1.17437000	-0.63586800
H	-2.10441700	-0.94799800	-1.88049800
H	-2.21804000	-2.57095800	-1.21151600
H	-0.72407400	-1.96445300	0.65388700
H	0.05655500	-1.57408800	-0.88024800
H	-3.14872700	1.05376100	-0.51058900

H	-3.94813600	0.70124200	1.01535300
H	-1.65162900	1.67007000	1.33511600
H	-1.79604500	0.06421200	2.05028400
H	-3.37394500	-1.70591900	0.80260500
C	0.71963700	0.29694700	1.11643500
C	2.01350400	0.22291000	0.31765400
H	0.70913800	-0.45466700	1.90923000
H	0.69767400	1.28316600	1.58742300
O	2.32309000	1.11348600	-0.45264200
O	-0.48678800	0.88680900	-0.95409800
O	-0.19153300	2.27691100	-0.66759400
H	0.78378600	2.23833900	-0.73227000
C	2.91467500	-0.97712500	0.53584100
H	2.30757500	-1.87758000	0.37577000
H	3.16893500	-0.99836600	1.60437900
C	4.16934000	-0.98564000	-0.33131200
H	4.77980000	-1.86555200	-0.11615600
H	3.91113800	-0.99556100	-1.39188300
H	4.77259800	-0.09389700	-0.15270000

R12-R

C	0.64197300	0.14896100	0.22522200
C	1.45126400	1.37459000	-0.21344200
C	2.82478700	1.02119900	-0.80460900
C	3.63129800	0.11887200	0.13712800
C	2.84243500	-1.14883300	0.48847000
C	1.46954400	-0.81382300	1.08724400
H	2.68456800	0.51269300	-1.76396300
H	0.86722700	1.95910500	-0.92907000
H	1.58357600	2.00343400	0.67493100
H	4.58794000	-0.14662600	-0.32269900
H	3.40327800	-1.76639500	1.19629200
H	0.88168600	-1.72156600	1.24691200
H	1.59879800	-0.34069700	2.06769900
H	3.37369800	1.94383400	-1.01602700
H	3.86845400	0.66891400	1.05748400
H	2.70601600	-1.75365700	-0.41426700
O	0.36017500	-0.55077000	-1.08390700
O	-0.42599000	-1.59490500	-0.97528400
C	-0.69625200	0.48680600	0.88779200
H	-0.47220200	1.00739900	1.82443300
H	-1.17372400	-0.45106600	1.16806000
C	-1.66700400	1.33026800	0.03366600
H	-1.49718900	1.15206200	-1.03206500

H	-1.48270800	2.39657000	0.20168800
C	-3.15021300	1.05002800	0.29884400
H	-3.36702300	0.94491000	1.36812300
O	-3.66407000	-0.07897100	-0.40465200
O	-3.19940400	-1.29265300	0.24533200
H	-2.45283600	-1.54481300	-0.32330300
H	-3.76224800	1.86588700	-0.09803900
TS			
C	-2.84042500	-0.62622700	-0.77729600
C	-1.72482600	-1.34106900	-0.00359700
C	-0.55394000	-0.41475100	0.35712300
C	-1.04777700	0.87432700	1.03658800
C	-2.16698200	1.57979300	0.25494700
C	-3.33141400	0.62666500	-0.04102600
H	-2.12499800	-1.75981400	0.92834500
H	-1.32934100	-2.17670800	-0.58699100
H	-2.46102700	-0.34157800	-1.76387100
H	-3.66927800	-1.32064400	-0.94613400
H	-1.41834200	0.59697000	2.03018700
H	-0.20439700	1.55340300	1.18999500
H	-2.51518100	2.44545600	0.82701500
H	-1.76531600	1.96575900	-0.68730200
H	-3.81100100	0.33296800	0.90246000
H	-4.09629600	1.13856800	-0.63317800
C	0.49508300	-1.16644300	1.20431200
H	0.59860700	-2.18132300	0.81023200
H	0.08757400	-1.26960000	2.21429500
C	1.89202800	-0.51940400	1.29657300
H	1.81809400	0.52049400	1.61673200
H	2.46490000	-1.05159600	2.06300400
C	2.69200300	-0.55576700	-0.00829200
H	1.82267900	-0.92767000	-0.91932900
O	0.06147600	0.07454200	-0.88463500
O	0.74991100	-0.97040900	-1.54142500
H	3.49913600	-1.28892100	-0.06573000
O	3.25529800	0.62374800	-0.45842100
O	2.32032100	1.72825200	-0.37475600
H	1.52564400	1.37243100	-0.82183300
P			
C	-2.19875400	-1.04300700	-1.03878300
C	-0.89880000	-1.37468800	-0.28966500
C	-0.21400300	-0.13348500	0.31796800
C	-1.21983000	0.67231300	1.16227200
C	-2.50129300	1.01462600	0.39404400

C	-3.17359200	-0.24478900	-0.16542500
H	-1.11038900	-2.07105500	0.53101700
H	-0.20754100	-1.88098700	-0.96932600
H	-1.95356700	-0.46197600	-1.93251000
H	-2.66413700	-1.97341100	-1.38047200
H	-1.46901000	0.07720100	2.04977700
H	-0.73015200	1.58378500	1.50910600
H	-3.18886000	1.55307700	1.05418100
H	-2.25191000	1.69405100	-0.42681200
H	-3.51945100	-0.87387200	0.66635300
H	-4.06343600	0.02277700	-0.74412100
C	1.01761900	-0.49510900	1.17799800
H	1.42380600	0.42899800	1.59844500
H	0.66781300	-1.09138400	2.02569000
C	2.14339900	-1.26195600	0.47452200
H	2.76463600	-1.78311500	1.21890300
H	1.77024200	-2.06707900	-0.16890000
C	3.12917700	-0.46296200	-0.33652400
H	3.88396600	-1.08045300	-0.86784100
O	0.18893000	0.61000700	-0.85463100
O	0.68144700	1.91744400	-0.47044700
H	1.64079600	1.73620800	-0.43695300
O	3.19258000	0.74274400	-0.41304400

R13-R

C	-0.93781600	-0.08791300	0.22952200
C	-1.57848600	-1.42173900	-0.17075600
C	-2.98308500	-1.26963200	-0.77486500
C	-3.90556900	-0.44994800	0.13545000
C	-3.29035800	0.92038200	0.44600500
C	-1.88906000	0.78666000	1.05752700
H	-2.90511300	-0.77881000	-1.75040400
H	-0.91932800	-1.94747800	-0.86640400
H	-1.63275500	-2.03390500	0.73725000
H	-4.88594900	-0.32635100	-0.33436700
H	-3.93091100	1.48243400	1.13213200
H	-1.42513200	1.76794700	1.18776000
H	-1.96129100	0.33322400	2.05303300
H	-3.40605000	-2.26233800	-0.95639900
H	-4.07465000	-0.99609600	1.07301800
H	-3.22855500	1.50802900	-0.47608200
O	-0.74163900	0.59977700	-1.10101700
O	-0.09270200	1.73717700	-1.02692300
C	0.42805700	-0.22770800	0.90765200

H	0.26569600	-0.74665600	1.85801300
H	0.77651900	0.77169400	1.16321300
C	1.50631500	-0.96278500	0.08453700
H	1.34663900	-0.80654600	-0.98656100
H	1.42848100	-2.04103800	0.25511200
C	2.95027500	-0.51993700	0.38013700
H	3.08084800	-0.30793600	1.44813700
O	3.31387700	0.66320300	-0.35169500
O	2.66936500	1.82194800	0.23677200
H	1.90682000	1.94460700	-0.35292500
C	3.97827200	-1.55210100	-0.07478000
H	4.98933600	-1.17765600	0.09178300
H	3.85105000	-2.48225700	0.48390300
H	3.86187600	-1.76939600	-1.13987500
TS			
C	-3.04591800	-0.87173100	-0.77229500
C	-1.85641800	-1.43276300	0.01828300
C	-0.80766700	-0.36512900	0.36500700
C	-1.45739400	0.86653900	1.01918400
C	-2.64824900	1.41805800	0.22003700
C	-3.68794800	0.32634000	-0.06160500
H	-2.20815600	-1.87996200	0.95654800
H	-1.35967500	-2.22470400	-0.54841500
H	-2.69756300	-0.56146300	-1.76258600
H	-3.78439100	-1.66375600	-0.93092200
H	-1.79848600	0.56415300	2.01604000
H	-0.70254600	1.64456500	1.16314300
H	-3.10153800	2.24618500	0.77382100
H	-2.28979100	1.83150600	-0.72796000
H	-4.13387200	-0.00545300	0.88578900
H	-4.50571800	0.73124800	-0.66586900
C	0.31917200	-0.96821600	1.23268400
H	0.54097800	-1.97469600	0.86603000
H	-0.07874100	-1.09251700	2.24417300
C	1.62804200	-0.15739800	1.30830800
H	1.42375000	0.88267700	1.56184300
H	2.24440900	-0.57009500	2.11510400
C	2.46166000	-0.18078800	0.01845600
H	1.62217900	-0.61658400	-0.87757500
O	-0.24924400	0.17283200	-0.88187000
O	0.56214800	-0.79276200	-1.51735600
O	2.89690600	1.05580500	-0.45502800
O	1.85396200	2.05922000	-0.40157800
H	1.11205700	1.62530000	-0.86706100

C	3.65320200	-1.11102400	-0.02509500
H	4.12801700	-1.09117100	-1.00734400
H	4.39515100	-0.81629900	0.72569500
H	3.33286900	-2.13301800	0.18842100

P

C	-2.27928200	-1.41739500	-0.86185000
C	-1.03900200	-1.40725900	0.04556200
C	-0.55589000	0.01404600	0.39804000
C	-1.72465900	0.85462000	0.94696900
C	-2.94540100	0.85346900	0.01959800
C	-3.41972000	-0.57339200	-0.28099900
H	-1.26209200	-1.92398300	0.98696300
H	-0.22980400	-1.96093500	-0.43899300
H	-2.00148900	-1.02400900	-1.84397700
H	-2.60640200	-2.45149300	-1.01280800
H	-2.00363500	0.44525100	1.92586400
H	-1.37244100	1.87450200	1.11011000
H	-3.75175600	1.43530300	0.47760300
H	-2.68132400	1.35751600	-0.91513900
H	-3.78486200	-1.03879000	0.64483100
H	-4.26566800	-0.55620700	-0.97559400
C	0.61197500	0.01674000	1.40845300
H	0.88921700	1.05521300	1.60752800
H	0.24007500	-0.39116700	2.35316900
C	1.86734000	-0.76149000	0.99968100
H	2.49685700	-0.91620300	1.88816800
H	1.63356500	-1.76956700	0.64577000
C	2.79058700	-0.08587600	-0.00292600
O	-0.11215100	0.52138100	-0.88262400
O	0.19904500	1.93183300	-0.78057700
H	1.15900300	1.88598700	-0.60075000
O	2.77123900	1.11145300	-0.21721900
C	3.79387300	-0.97906700	-0.69872800
H	3.27302600	-1.54091800	-1.48193000
H	4.57449500	-0.37471500	-1.15861900
H	4.22924200	-1.70948300	-0.01163800

OH

O	0.00000000	0.00000000	0.10835600
H	0.00000000	0.00000000	-0.86685200
